# Assessing the feasibility and acceptability of a school-based non-pharmacological intervention for ADHD: the Flex toolkit

**DOI:** 10.1186/s40814-026-01879-7

**Published:** 2026-07-22

**Authors:** Abigail Emma Russell, Suzie Holt, Charlotte Kelman, Tamsin Ford, Darren Moore, Rachel Hayes, Judi Kidger, Edmund Sonuga-Barke, Barney Dunn, Linda Pfiffner, Eleanor Bryant, Rebecca Gudka, Kirsty Cordwell, the Tools for Schools Planning Group

**Affiliations:** 1 University of Exeter Medical School, St Luke’s Campus, Exeter, UK; 2 Department of Psychiatry, University of Cambridge, Cambridge, UK; 3 Graduate School of Education, University of Exeter, Exeter, UK; 4 Centre for Public Health, University of Bristol, Bristol, UK; 5 Institute of Psychiatry, Psychology and Neuroscience, King’s College London, London, UK; 6 Department of Psychology, University of Exeter, Exeter, UK; 7 Department of Psychiatry and Behavioral Sciences, University of California San Francisco, San Francisco, USA

**Keywords:** ADHD, School-based intervention, Behaviour change, Feasibility, Acceptability

## Abstract

**Background:**

Attention deficit/hyperactivity disorder (ADHD) is a prevalent and impairing neurodevelopmental disorder. Traits cause impairment for 5–10% of children. School is a particular challenge for children with ADHD, who are at risk of exclusion, mental health problems and poor attainment. Existing school-based interventions have evidence of effectiveness, however are not implementable in UK mainstream school contexts due to resource constraints. We co-designed an individualised novel non-pharmacological intervention for ADHD (the Flex toolkit) based on principles of behaviour change, using Intervention Mapping. We aimed to assess the feasibility and acceptability of the Flex toolkit, and assess whether a definitive evaluation in schools is feasible.

**Methods:**

A multiple baseline case series study was conducted with eight UK primary schools. Teachers, parents and children completed both quantitative and qualitative measures over 1 year, with the intervention being used for approximately 16 weeks. Mixed methods analysis was used to assess feasibility and acceptability, and evidence for perceived impact of the intervention on relevant outcomes as well as mechanisms of action from the intervention logic model was assessed.

**Results:**

Forty children with ADHD, 53 teachers and 49 parents/caregivers participated. The intervention was deemed to be highly acceptable and useful; however, it was not feasible for staff to implement with fidelity in schools as an unguided intervention. Those who engaged with the intervention often perceived it to have beneficial impacts for study children as evidenced in qualitative data. The research design was considered to be both acceptable and feasible with some recommendations to streamline procedures for a definitive trial.

**Conclusions:**

The Flex toolkit is acceptable to schools. It was perceived to improve a range of outcomes that relate to dimensions of mental health and school-based impairment; however, the study was not powered to detect quantitative change. A definitive evaluation would need to test a supported delivery model of the intervention to ensure that it is delivered with fidelity; we recommend a “coach” whose role would be to provide light-touch support to teachers to select and implement intervention components. The research design for a definitive evaluation can mirror many of the aspects of the feasibility study design.

**Supplementary Information:**

The online version contains supplementary material available at 10.1186/s40814-026-01879-7.

## Key messages regarding feasibility

- Existing school-based interventions for ADHD have not been implementable in UK school contexts. We assessed whether a novel, individualised behaviour change intervention to support children age 4–10 with ADHD would be feasible and acceptable in terms of intervention content and delivery, and research design and process for a definitive evaluation.
- We found that the intervention was acceptable to school staff, children and parents. However, in an unguided format, it was not feasible to deliver with high fidelity. We found that the research design was both feasible and acceptable.
- A definitive evaluation would need to test a supported delivery model of the intervention to ensure that it is delivered with fidelity; we recommend a “coach” whose role would be to provide light-touch support to teachers to select and implement intervention components. Minimal change would need to be made to the intervention content itself. The research design for a definitive evaluation can mirror many of the aspects of the feasibility study design, with reduction in the frequency of measures collected.

## Background

Attention deficit hyperactivity disorder (ADHD) is a prevalent and impairing neurodevelopmental disorder, with core symptoms of inattention, impulsivity and hyperactivity [1]. Around 5% of young people in the UK meet diagnostic thresholds, although rates of diagnosis are lower [2]. The core symptoms are dimensional traits, so children with sub-clinical levels also experience impairment [3, 4]. For many, ADHD is associated with negative health and functional outcomes [5–11]. School can be a particular challenge for children with ADHD, as social, behavioural and academic challenges can make engaging with and meeting expectations of the school environment difficult [8, 9]. Trajectories of difficulties often begin in primary school [12]. Regardless of diagnostic status, early intervention that focusses on individual strengths and challenges is likely to be a promising avenue of intervention [5].

Existing school-based interventions have a moderate evidence base, with multiple randomised controlled trials (RCTs) finding beneficial effects of (largely multicomponent) interventions [13]. Remaining challenges are twofold: firstly understanding which components generate impactful change for children with particular traits. Micro-trials of antecedent- and consequence-based behavioural interventions have evidenced effective reductions of problem behaviours for both intervention types [14]. Secondly, many of the best-evidenced interventions are resource-intensive and can present feasibility challenges when delivered in mainstream school contexts, where there are limited staff and budgets [13]. This highlights a clear and urgent need for a low-cost school-based intervention for ADHD and associated behavioural traits, along with evidence of which components of the intervention are associated with efficacy for which children.

Previous research has identified what a low-cost school-based intervention should target to have the biggest impact [13, 15]. Taking a neuroaffirmative approach, it is important to consider changing the environment around the child as well as interpersonal relationships, as opposed to addressing symptoms of ADHD “within” the child as the key areas of focus [16].

### The Flex toolkit

To address the gaps identified above, we have developed a novel intervention: the Flex toolkit (described and shown in Supplementary Material S1).

The toolkit includes a range of tools for different individual needs and is an individualisable multicomponent complex intervention hosted online. In the first prototype, these comprised four core components: a strengths-based “Know Me” child activity, along with “Know ADHD” (a series of short psychoeducation videos explaining scientific concepts underpinning the aetiology and understanding of ADHD), a functional behaviour analysis-based “Setting Goals and Targets” exercise, and a digital “Daily Report Card” to facilitate regular, positive, home-school communication. Key to the toolkit are individualised strategies that follow these components, with teachers, parents and Special Educational Needs Co-ordinators (SENCos) identifying two specific goals for their student, and selecting two strategies from the six modules and ~ 40 available options, to implement with the student.

**Fig. 1:**
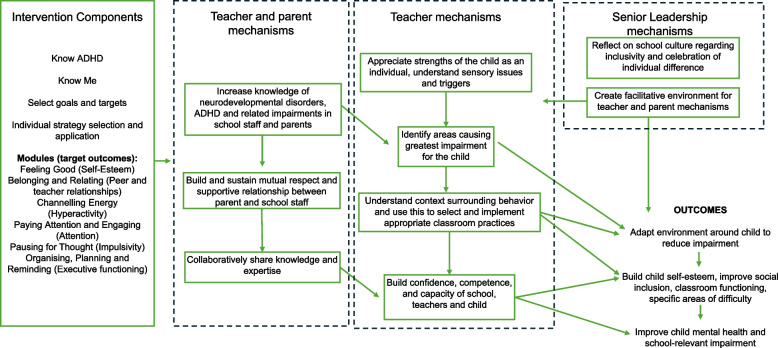
Flex toolkit theory of change

In this paper, we describe the evaluation of the Flex toolkit’s feasibility and acceptability in mainstream UK primary schools. We focus on both the feasibility and acceptability to staff delivering the intervention in line with the underpinning logic model (Fig 1), acceptability of the intervention to parents and children, as well as the feasibility of conducting a fully powered rigorously designed definitive evaluation. We used the definition of acceptability proposed by Sekhon et al. [17]: “a multi-faceted construct that reflects the extent to which people delivering or receiving a healthcare intervention consider it to be appropriate, based on anticipated or experienced cognitive and emotional responses to the intervention”. We use the term feasibility in the context suggested by Eldridge et al. [18]: “whether something can be done, should we proceed with it, and if so, how”. In this study, feasibility relates both to the intervention delivery and the design of a definitive trial. A further relevant concept for this study is implementation fidelity: the “degree to which an intervention is delivered as intended” [19].

We aimed to:

1. Assess whether the Flex toolkit is feasible and acceptable to implement in the school setting.
2. Establish whether a future definitive trial of effectiveness is feasible.

Our secondary aims were to:

1. Identify suitable outcome measures to assess core ADHD symptoms, child and teacher well-being and academic progress, and identify which of these would be the primary outcome of a future definitive trial.
2. Assess whether classroom functioning, ADHD symptoms or other aspects of mental health were perceived to improve following use of the toolkit.

## Methods

### Study design

**Fig. 2:**
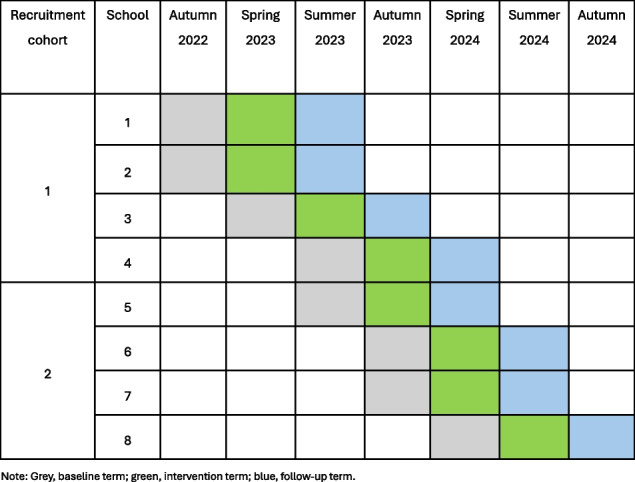
Case series design

We conducted a randomised iterative mixed-methods case series study from 09/2022 to 12/2024. Participants were clustered within schools, with schools as the units of randomisation, randomised to the time (school term) when they used the toolkit. All schools/children participated in the study for one calendar year, comprising a baseline school term, an intervention term and a follow-up term, with clusters staggered in terms of start date. The study protocol was published [20]. This staggered design was selected to enable revisions of the intervention throughout the study, to maximise the changes of meeting the study aims [21], and randomisation to using the toolkit in different school terms allows for inferences to be made as to whether the intervention is improving outcomes, with consistent improvement following the introduction of the core components enabling researchers to potentially rule out alternative explanations for behaviour change, such as improvement due to other support in place or differences in behaviour across the school year. The design is a case-series as opposed to stepped-wedge, as each school started at a unique point in the study but followed the same schedule, as opposed to all schools starting the study at the same calendar time and having the intervention introduced at different points (Fig. 2) [21, 22].

### School sample

The target sample was eight primary schools in the South West of England. Schools were recruited via opportunistic sampling (see Supplementary Material S2). Eligible schools were mainstream primary provision (ages 5–11 years), with at least one female student meeting student eligibility criteria, to address the sex bias often present in ADHD research and treatment [23].

### Population

The planned sample size was 16–32 children, along with their teachers, teaching assistants (TAs), school SENCos and headteachers. The aim of the study was not to assess effectiveness/efficacy; the sample size was planned to generate sufficient variability in participating students and teachers to test our feasibility study aims. We estimated 4–8 eligible children per school using a conservative estimate of 3% prevalence, and planned to recruit 50% of those eligible. Ethical approval was provided by the University of Exeter College of Medicine and Health Research Ethics Committee (Jan22/B/300).

### Sample size justification

Sample size was planned based on the main aim of the study: to assess whether the Flex toolkit is feasible and acceptable to implement in the school setting, using a case-series design allowing iteration of the toolkit across schools. A sample of eight schools allowed for up to five successive iterations of the toolkit (Fig. 2) to have the best chance of achieving this aim, and to obtain qualitative data from a sufficiently varied and diverse sample of teachers, children and families [24]. This sample size also allowed for varying the intervention term across the school year to inform our second main aim regarding the design for a fully powered RCT, and to ensure purposive representation of schools from areas of high and low socioeconomic disadvantage. Our median sample was larger than that in existing feasibility cluster-RCTs (7.5 schools), and as it was non-randomised, we were able to obtain data relevant to our aims from all schools [25].

### Recruitment procedure

Schools were recruited in two cohorts of four in order to minimise delay into study entry, then randomised to the order in which they started in the study (Fig. 2). Each participated in data collection for one calendar year. To ascertain when the intervention delivery would best be situated in a subsequent trial, schools began the intervention at the start of one school term in either September, January or April/May. Schools had a one-term baseline term, an intervention term where the toolkit was introduced, and then a follow-up term where the school was free to continue or cease using the intervention: several chose to continue. Data collection included a range of quantitative and qualitative measures. Quantitative data were collected across all three terms, with qualitative data collected in intervention and follow-up terms. Eligibility screening and recruitment are described in the Supplementary Material S3.

### Feasibility and acceptability outcomes

Feasibility and acceptability of the study design and intervention were assessed against study progression criteria (Table 1) using a combination of quantitative indicators and analysis of qualitative data.

**Table 1:** Study progression criteria

	Green - acceptable	Amber - discuss, modify	Red - redesign	Assessing
Recruitment of schools	6 or more	5	4 or fewer	Feasibility
Recruitment- teachers, children, parents	> 65%	20–65%	< 20%	Feasibility
Retention of schools, teachers, children and parents in study	> 65%	40–65%	< 40%	Feasibility
Training completed (teachers)	> 90%	70–90%	< 70%	Acceptability and feasibility
Introductory video watched (parents)	> 50%	20–50%	< 20%	Acceptability
Child strengths activity completed	> 50%	20–50%	< 20%	Acceptability
Adherence to digital daily report card	> 70%	50–70%	< 50%	Acceptability
Teacher-completed measures	> 70%	50–70%	< 50%	Feasibility
Parent-completed measures	> 50%	20–50%	< 20%	Feasibility
Child-completed measures	> 70%	50–70%	< 50%	Feasibility
Observational measures	> 50%	20–50%	< 20%	Feasibility
Attendance at toolkit-related meetings	> 75%	40–75%	< 40%	Acceptability
Percentage of occasions toolkit reportedly used as instructed	> 75%	50–75%	< 50%	Acceptability and feasibility
Follow-up measures completed	> 50%	20–50%	N/A	Feasibility

### Quantitative measures

Table 2 shows the data collection schedule; intensive repeated measures are a feature of the case series design selected along with input on data collection schedule from the planning group [26]. Measures included ADHD symptoms using the Strengths and Weaknesses of ADHD Symptoms and Normal‐Behavior (SWAN) Questionnaire, scored from − 3 to + 3 with higher scores indicating more severe symptoms [27], and classroom behaviour: measured using the classroom functioning problem behaviour subscale from the Social Skills Improvement System (SSiS) [28] that captures social, academic and competing problem behaviours in the classroom environment, reported by parents and teachers.

**Table 2:**
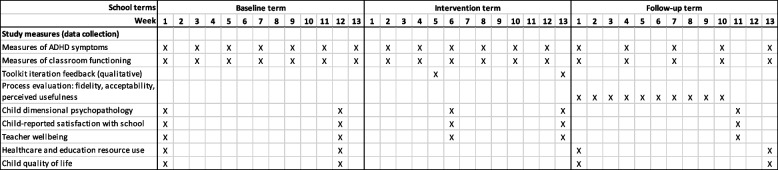
Data collection schedule

Children reported on satisfaction with school using the How I Feel About My School questionnaire [29]. Child health-related quality of life was measured using the parent-proxy and child-completed Child Health Utility 9D (CHU9D) [30]. Observations of child behaviour were conducted using the Scope Classroom Observation Checklist [31]. All researchers conducting observations were trained to be reliable with CK and SH: based on a two-way mixed-effects intraclass correlation coefficient model ICC = 0.91 (95% CI 0.87–0.94); methods reported elsewhere [32]). Teacher–child relationship was teacher-reported using the UK version of the Student–Teacher Relationship scale [33].

Child dimensional psychopathology was assessed using the teacher and parent-reported Strengths and Difficulties Questionnaire [34]. Teacher well-being, efficacy and burnout was measured using the Warwick‐Edinburgh Mental Well-being Scale 14-item Teacher Survey [35] and the Maslach Burnout Inventory-General Survey [36]. Child social skills and academic competence were measured with the pertinent subscales from the SSiS [28].

Child health and social care and education resource use was parent-reported using a bespoke tool designed through drawing on the Client Service Receipt Inventory [37] and measures in the Database of Instruments for Resource Use Management Repository. The finalised measure is included in Supplementary Material S4.

### Qualitative measures

All school staff were invited to two telephone or in-person process evaluation “toolkit iteration interviews” at their school during the intervention term. Staff were subsequently invited to a process evaluation focus group close to the end of follow-up. All parents were invited to brief telephone interviews during the intervention terms. Half of parents and all children were invited to interview at the end of the follow-up term.

All interviews and focus groups followed semi-structured topic guides co-developed with the planning group. Interviews were an opportunity for teachers to discuss what aspects of the toolkit they had engaged with, and what changes they considered were important in order for the team to meet their aims of having a feasible, acceptable intervention by study end. Children’s interviews took place at home or at school based on parent preference, and questions were asked while the child and researcher engaged in activities or games togther.

### Procedure

Parents and teachers completed questionnaires online, with reminders sent by email (teachers, parents), text and phone call (parents). Paper copies were made available, and researchers were available to complete parent measures over the phone or in person as a structured interview where preferred.

### Baseline

During baseline, teachers and parents completed measures on repeated occasions. Members of the research team observed each child in the classroom once per half-term and completed child-report measures in school 1:1 with children. Children were asked for assent, and if they indicated disinterest or discomfort were asked if they wished to continue or if they would prefer to return to class. They received stickers to thank them for their time. Two to three weeks before the end of baseline, teachers received individual login details for the Flex toolkit and a 1:1 introduction lasting approximately 15 min with a member of the research team. They were made aware that the purpose of the study was to see how they used the toolkit and provide feedback on this to the research team.

### Intervention

At the start of the intervention term, researchers held a drop-in session with teachers on a non-pupil day so they could ask questions about the study or clarify use of the toolkit. Teachers were emailed by the study team outlining the first and next steps of intervention use, every 3–4 weeks during the intervention term. Data collection continued as above. In addition, process evaluation interviews were conducted with the teachers, in-person, on the phone or via MS Teams. These were scheduled at the end of each half-term, to give teachers as much time as possible to engage with the toolkit prior to providing feedback.

### Follow-up

At the start of the follow-up term, teachers were informed that they could continue to use the intervention if they wished. Quantitative data collection continued and focus groups were held with all participating school staff to gain final feedback on the toolkit, research data collection and other points of interest to the study aims.

### Incentive

Teachers, children and primary caregivers each received a £10 voucher to thank them for their involvement. Schools were paid ~ £700 (scaled relative to the number of children successfully enrolled in the study) to cover staff time.

### Analysis

Mixed-methods analyses were undertaken, integrating insights from synthesis of qualitative data with quantitative metrics as described in the protocol [20]. Qualitative data from toolkit iteration interviews and process evaluation interviews/focus groups was audio recorded, transcribed and analysed using the framework method and thematic analysis [38] as per the protocol.

Feasibility and acceptability of the study design were assessed against study progression criteria and reported in relation to recruitment and retention of participants and completion of study measures.

Acceptability of the intervention was quantitatively assessed against the progression criteria that relate to completion of each component of the toolkit, attendance at toolkit-related meetings and the percentage of occasions the participant reports using the toolkit as instructed. Qualitative findings were used to explain and further explore quantitative results in a mixed-methods synthesis. Modifications were made iteratively as findings emerged. Where indicators were “green”, this indicated acceptability. In addition, feasibility, acceptability and perceived usefulness of the intervention was assessed through synthesising responses relating to each component of the toolkit in a framework: specifically to assess whether the components and the overall toolkit were considered appropriate to support children with ADHD in school. Data on adverse events was captured in all qualitative data collection incidents; none were reported.

When asked directly about outcomes for a future trial, teachers and parents often struggled to articulate one outcome; instead, analysis to address this aim was based on parent, teacher and child perceived impacts on behaviour due to the toolkit use, identified from qualitative data.

To assess evidence of impact on the quantitative outcomes, mixed effect multilevel models were run in Stata v17 using the *mixed* command, accounting for multiple observations per individual and with a random slope to allow individual trajectories to vary over time. Missing data were handled using maximum likelihood estimation, with all available observed data utilised. We did not cluster by school for this exploratory analysis as all children received a unique intervention, even if part of the same school. This was to assess whether there was evidence of change in the SWAN, SISS problem behaviour, SDQ total difficulties and SDQ impact subscales between the baseline, intervention and follow-up terms (accounting for repeated measures per child as fixed effects, and trajectories over time as random effects). This analysis was exploratory as the study was not powered to detect quantitative change. Further findings from the quantitative measures will be reported in a subsequent publication; the focus herein is on whether these measures were feasible and acceptable in terms of data collection from participants.

## Results

### Study sample

Thirty-four children were recruited and retained throughout the study. Demographic data were available for 33 of these children (Table 3). Two-thirds of participating children were male, the mean age was 7.7 years and the majority were of white British ethnicity (reflective of the geographic area of the study [39]). One-third had received a clinical diagnosis of ADHD, and half of these were taking medication. One in five had a co-occurring condition, most commonly autism spectrum conditions.

**Table 3:** Child demographic characteristics (*n* = 33)

Characteristic	% or mean (SD)
Sex (% male)	66.7
Ethnicity (% white British)	93.93
Age (mean (SD), years)	7.67 (1.88)
Co-occurring conditions (% yes)	21.2
ADHD diagnosis (%)	33.3
Age of diagnosis (mean, years)	7.75 (0.96)
% diagnosed taking ADHD medication	54.5

### Feasibility and acceptability of the intervention

#### Adherence to intervention delivery

Continuation criteria for the toolkit engagement are shown in Table 4. Given the iterative nature of the study and intervention, the prototype was amended based on feedback at two points during the study—as such, some criteria in the protocol became invalid (e.g., attendance at toolkit-related meetings). The most pertinent feasibility and acceptability metrics were therefore as follows: training completed (teachers); introductory video watched; parents and teachers; child strengths activity completed; and percentage of occasions toolkit reportedly used as instructed—defined as teachers reported using at least one strategy with the study child during the intervention term.

**Table 4:** Toolkit components delivered

Toolkit component	*N* teachers reporting use with study child	Percentage of teachers using with study child (*N* children = 29)	Continuation criteria status (red/amber/green)
Know ADHD	9	31.0	R
Know Me	16	55.2	A
Strategy 1	25	86.2	G
Strategy 2	12	41.4	R
Strategy 3	3	10.3	R
Overall number of “toolkit users” (at least one component)	27	93.1	G
Mean “dose” (scored 1 per component, range 0–4)	2.14		

Training was delivered to all teachers participating in the study. Data on toolkit component and strategy use were available for 29 children whose teachers/TAs participated in the interviews. Components were coded as present where participants mentioned engaging with them. Only one teacher reported using the daily report card. All but two teachers reported engaging with the toolkit in some manner, indicating high adoption rates. Quality of implementation was not able to be assessed based on the data collected. In terms of intensity, on average, teachers delivered 2.14 components to the child, with Know Me, and at least one strategy being most frequently used. Twelve of these teachers used two strategies, and three teachers used three. The Know ADHD videos were mentioned in nine process evaluations as being watched by teachers.

Data from YouTube showed that the nine videos in the Know ADHD section have been viewed a total of 774 times, and the learning snippets within the toolkit a total of 578 times (as of 24/6/25), indicating good uptake across the study. Overall, the quantitative data suggest that fidelity to individual components of the intervention varied widely based on the pre-published continuation criteria; however, > 90% engaged with the toolkit in some way, with 86% applying at least one individualised strategy with their student.

#### Feasibility and acceptability of intervention components and strategies

##### Know ADHD

Teachers reported that the toolkit increased the profile of children with ADHD within the class and school, and appreciated the variation of presentations of ADHD more than prior to the intervention. The Know ADHD videos were reported to be “*informative*”:

“It was useful to have those videos from like the point of view of a parent that has a child with ADHD to watch it and see what…life was for their child in school”

In some cases, parents reported teachers recommending they watch the Know ADHD videos, as they were “*really helpful*”.

Parents also found the videos useful when they engaged with them: “*it wasn’t until we started doing Tools for Schools … I’d never watched any videos or anything, but since sitting down and watching videos on how to help them and understand them better, it’s really helped.*”

##### Know Me

Teachers who completed the Know Me activity often reported gaining additional insight into the child: “*It was having that time to really get her voice which doesn’t always happen in a very busy classroom. So I found it really useful. A couple of things came up that I didn’t necessarily know about straightaway. She always comes across in class as very calm but actually hearing more what she was talking about, there was a lot more going on in there. So that was really useful to kind of get to know her and what’s going on inside a bit more.*”

“That was a perfectly worthwhile activity… think he found it enjoyable. I found it was useful to be able to do”

For others, the activity highlighted aspects beyond strengths, such as the way a child believed they were perceived by others: “*one of the questions something like ‘what is it that you want other people to know about me?’, and she’d said something like ‘I’m actually quite a nice person’…and ‘because I hurt people, doesn’t mean I’m unkind’… it’s certainly helped me to get a better picture of perhaps some of her feelings.*”

Others reported using their knowledge of the child gained through the activity to tailor their relational approaches, and engage the child with behaviour change.

##### Setting goals and targets and use of the Behaviour Web

Teachers did not often implement the guidance for setting goals and targets; many stated that this was part of their usual practice, or that goals were obvious for the child so the need for this to be a formal step was unclear: “*With [child] it was quite clear that how he came across in the classroom was a big thing, his disruption and inability to focus, and that was apparent*”. Revisions to the toolkit over the study removed the requirement for functional behaviour analysis.

Teachers reported instead using the drop-down Behaviour Web, a routing feature of the website, to select problems that were relevant to the child: “*as I got to know [child] and work out the times in the day that were trickier for him, which was lunchtime,…then used that to then look through for specific targets that would help him*”.

“I like the way that on your toolkit, it says something like, “What is the problem?” And you’ve got a drop-down menu, haven’t you, and then it suggests, “These are potential tools that might work for this child.” I find that really helpful. That was a good way of finding the tool that I thought would work for her.”

##### Digital daily report card and home-school communication

This was infrequently used and therefore was presented as optional to schools 4–8. Teachers had concerns about duplication with existing home-school communication strategies: “*We have quite a lot of layers because we have house points, we have dojos, so it’s just…another layer…*”.

“I’m very guilty of not doing at all is the home school diary bit online and I can see again that that would be useful but it’s so much easier to catch the parent at the end of the day”

Communication between school and home varied widely across study participants, often described by parents as a challenge. Teachers also described families sometimes being “*very difficult to get engaged*” or as “*a family that struggles with keeping on top of things*”. Further detail is provided in Supplementary Material S5. A few school staff described their involvement in the study as improving school-home relationships, one teacher described how it had been “*useful for [mum] to realise the things that we are putting in place for him and also just keeping it positive*”.

##### Use of specific strategies and impacts on behaviour at school

Strategies containing movement opportunities, emotion-regulation activities and specific labelled praise were most frequently selected; a wide range of the strategies offered in the toolkit were reported to have been considered or tried by teachers. Overall, strategies used were perceived to have resulted in behaviour improvement. Participants mentioned a range of factors that had changed for them or the study child during the use of the intervention. Further detail on these is provided in the Supplementary Material.

Teachers perceived improvements in their relationship with the child. Teachers reported that a better understanding of the child, and increased awareness and understanding of ADHD, meant that they could make learning expectations appropriate to their level, and explicit to the child, which was motivating for the child in class.

Relating to this increased attention and focus on the child, teachers reported that they were better able to support them, using positive reinforcement. The “*carrots*” of positive reinforcement were considered very helpful in supporting the child to engage with work:

“I was catching her out doing anything good, like sitting still for a couple of minutes, or putting her hand up, or engaging, or anything at all that she was doing that was positive I was praising her for it, so that was obviously really good, and I think also then that did happen with the rest of the class, the positivity spread a little bit, so that was really good. But yes, I do think it was beneficial for her”

Teachers reported identifying and scaffolding opportunities for the child to self-regulate, interacting with the child in a different manner and so becoming less angry or negative in their attitude toward the child, and increasing the quality time they spend together. Teachers reporting a better understanding of what a child was thinking and feeling. With some strategies, teachers reported giving the child choice and increasing independence with the use of regulation strategies such as the Box of Tricks: “*…those are the ones he chose himself. So I’m thinking because he chose them, they’re working more so than if I’d chosen them*”.

Some teachers reported benefits for their whole class: “*actually the impact it is for them that it has on the whole class and…attention and listening*”.

Teachers’ experiences with the toolkit were that there were often clear benefits for the child in classroom. This included an increased willingness to do academic work, often supported by the child being more able to stay physically within the classroom setting for longer periods of time. Peer relationships were also reported to improve for some children; teachers reported that children were better able to make “*good choices*” and became “*less reactive*”, leading to fewer problems with friendships. They also reported strategies supported children to transition out of a negative mood, for example, if they did have problems with peer interactions, and children seeking adult support for help with peer interactions. Other teachers reported the benefits of movement strategies for the child, mentioning multiple impacts, most commonly the idea that the child could “*reset*”.

“I introduced the movement mat at the start of the year, and that definitely helped [child’s] focus and engagement in the lessons, and I’ve found now he’s more independent with knowing when he needs to go and do something or he needs something to fiddle with, and he’ll do that quietly without making a scene”

Movement, and other strategies, were reported to promote the child’s engagement with learning and academic subjects. This included increased organisation skills, better ability to focus and children’s willingness to try with work. Benefits were reported for different children in reading, maths and the presentation and completeness of work.

##### Time for toolkit use

The main barrier to engagement with the study at school-level was the time made available to teachers, which was very little, in spite of schools receiving incentive to participate of approximately £200 per child. Teachers believed that allocated time to understand the toolkit and prepare for implementing the strategies was critical, and most reported that this was not provided by their school. Challenges of finding cover for staff were substantive: teachers reported that even if supply or cover from senior leadership or the SENCo had been arranged, sickness absence of other staff on the day could result in none being available. Teachers thought that this time was critical for their ability to deliver the intervention with fidelity, citing 30–60 min needed to become familiar with navigating and using the toolkit. Teachers proposed that half-day group training on the intervention would be helpful (rather than 1:1 walkthroughs).

There were a range of suggestions as to how best to overcome this resource barrier: the school or trust mandating use of the toolkit, using time within staff meetings (either all staff or the study teachers being excused at this time), or allocating teachers time out from the class (in addition to mandated time for Planning Preparation and Assessment and Early Career Teacher time). Teachers reported that time out of class was legitimised by the study researcher being in the school, and with two of the later schools in the study, the researcher came in for an additional visit between process evaluations for 15-min 1:1 sessions so that teachers had opportunity to navigate the site and select a strategy, and print any resources needed. This was very positively received: “*talking with you really helped me*”. Teachers reported enjoying having a conversation about the toolkit as opposed to the video walkthroughs (although these were also perceived as useful); the critical challenge with the video walkthrough was that teachers were not able to directly ask questions.

This ‘ask’ for more time and input from the study team in terms of toolkit delivery was noted across schools. Overall, teachers reported that the walkthrough was useful, but some struggled to recall this within a week or two, and additional familiarisation time was suggested, either at the point when the toolkit was being implemented, or as a practice run in the preceding school term in order to allow them to become used to navigation of the site. They proposed a model whereby they had access to someone who would check in every week or two, ensuring that they knew what to do next and had the time to prepare to implement this. Teachers thought that this “coach” could be the intervention developer, training the SENCos in toolkit use, or having access to an external expert in mental health.

Opinions varied as to whether an expert on the toolkit within-school was more use than an external expert: external experts coming to the school would increase the need for senior leadership team buy-in and allow for teachers to be released from class time, but internal expertise would allow in-the-moment problem solving and consultation, and access to advice and recommendations. SENCos were perceived to know the children well, know about ADHD and be able to hold teachers accountable within the existing structures of the school. However, SENCos in some schools were perceived to be too busy to hold this role, or not available to staff. Teachers also reported wanting explicit reminders of expectations regarding the use of the toolkit each month. The study team sent emails once per half term, and these were positively received but considered less memorable than the suggested newsletter or highlights email. These need to be sent at a separate time to data collection emails. Teachers reported that they found definitive deadlines helpful, and suggested using email calendar invites for this purpose. Teachers often reported being unaware of which colleagues were also part of the study. They perceived that more within-school collaboration would have been beneficial.

### Feasibility of a definitive evaluation

#### Recruitment and retention

All recruitment and retention data met the “green” continuation criteria (Table 5). Nine schools were recruited, of which eight were retained. One school withdrew between school consent and identification of relevant students, due to staffing capacity. Forty children were recruited of which 34 were retained (85%). The six students who withdrew all did so because they left their school during the study for reasons unrelated to study involvement. Retention rates for parents and guardians matched this, with 41 of the 49 recruited retained. Similarly high retention rates for headteachers and SENCos (80%; 77%) reflected staff transition out of schools. A small number of TAs were recruited, with 100% retention. Of the 53 recruited teachers, 47 remained in the study (89%). Those who withdrew did so for a range of reasons; some withdrew due to a combination of the research study burden and lack of allowance or support for their engagement from their school. Others were due to long-term sick leave and leaving the school. Notably, all teachers who withdrew gave permission for student data collection to continue. Withdrawals were distributed across the study schools.

**Table 5:** Feasibility of school and individual recruitment and retention

	*N* recruited	*N* retained	*N* withdrawn	Percentage	Continuation criteria status **(red/amber/green)**
Schools recruited	9	8	1	88.9	G
Teachers recruited	53	47	6	88.7	G
SENCos	13	10	3	76.9	G
Headteachers	10	8	2	80.0	G
TAs	6	6	0	100.0	G
Other school staff	1	1	0	100.0	NA
Children	40	34	6	85.0	G
Parents/guardians	49	41	8	83.7	G
Recruitment overall	172	147	25	85.5	G

#### Completion of study measures

All study measure metrics met “green” continuation criteria (Table 6). Using data including all recruited students and teachers (up to point of withdrawal if withdrew), completion of questionnaire measures met “green” criteria.

**Table 6:** Feasibility of completion of research measures

	Mean completed (%)	Mean completed (*N*)	Continuation criteria status **(red/amber/green)**
Teacher	86%	10	G
Parent	78%	4.3	G
Child reported	98%	8.8	G
Child observed	98%	8.8	G
Process evaluation (teachers)	78%	7.1**	G
Focus group (teachers)	76%	4.3**	G
Process evaluation (parents)	54%	-	G
Follow up interviews (parents)	57%	-	G
Follow up interviews (children)	80%	-	G

**Mean refers to mean number of staff per school. Note that some of the data includes teachers who are job-sharing, so actual coverage per child is higher than the statistics here

Collection of qualitative data from school staff was also in the “green” range. Process evaluation data was collected on 78% of planned instances (two per teacher, during the intervention term), and 75% of eligible teachers participated in focus groups (end of follow-up term). In addition, qualitative data were captured from SENCos on seven occasions (mix of focus groups and interview), with SENCos from five schools. SENCos from three schools did not participate in qualitative data collection. Over 50% of parent interviews were completed as planned, and 80% of child interviews, within the “green” continuation range. The research team did not consistently approach parents and children in schools 1 and 2, and if these data are excluded, then completion rates were higher: 76% for parents during the intervention term, 68% in follow-up term and child completion was 92.6%. Parent-completed qualitative interviews were usually with mothers; however, six fathers also participated.

#### Additional school-level considerations for a trial

There were a range of structural and system-level factors that impacted on the delivery of the study within schools. These are discussed in the Supplementary Material S5 in depth, but in brief include recommendations to increase teacher involvement in choice of students, ensuring that supply cover is provided for teachers to be able to engage with the toolkit fully, in consideration of the Special Educational Needs and Disabilities (SEND) culture of participating schools, involvement of a larger number and breadth of children and school staff, building in collaboration across teachers delivering the intervention within a school, and potentially providing feedback to teachers about child observations.

#### Suitability of outcome measures

The study design and conduct were positively received, indicating acceptability. Some of these were practical aspects: teachers and parents enjoyed the online format of questionnaires, although some flexibility in format was required (e.g., breaking long questionnaires into shorter chunks for some schools, paper or phone format for some parents and teachers); as such, some teachers did not complete all the study measures. The questions were considered to be appropriate, but repetitive. Teachers frequently asked whether they could see the exact scores they had given previously: they believed that they were better able to respond to questions based on change than standardised measures. Indeed, some suggested that we simply ask “has anything changed for this child? If so, what?” Further comments asked for additional free text boxes, as teachers thought that contextual factors such as bereavement could explain negative changes in children’s behaviour. Teachers thought that the short questionnaires (SWAN and SSiS problem behaviours (PB)) would not capture the behaviour changes that they perceived were being caused by the toolkit (suggesting different primary outcome measures would be required for a definitive trial). Some wanted to be asked instead if they were seeing any changes in the child proximal to the strategy they were implementing.

**Fig. 3:**
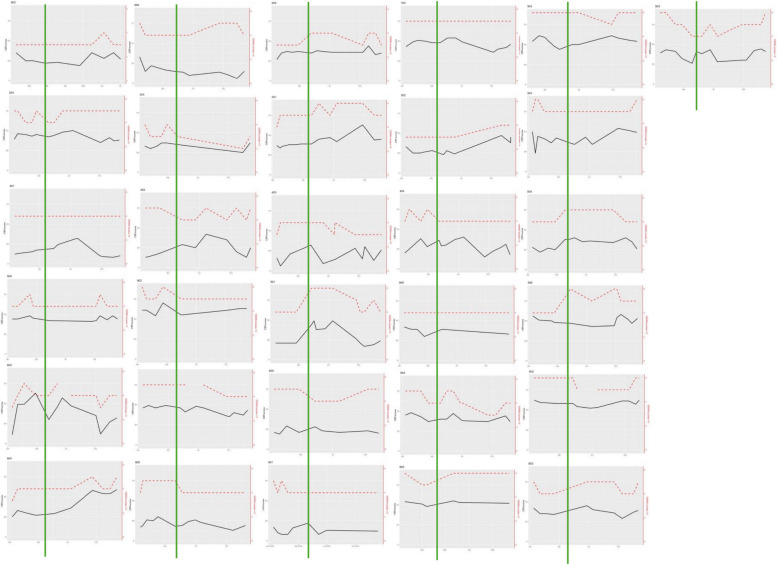
Graphical representations of child scores on SWAN and SISS-PB over time Red dotted lines are SWAN scores and black solid lines are SISS-PB scores. The green horizontal line represents approximately when the intervention was introduced

**Fig. 4:**
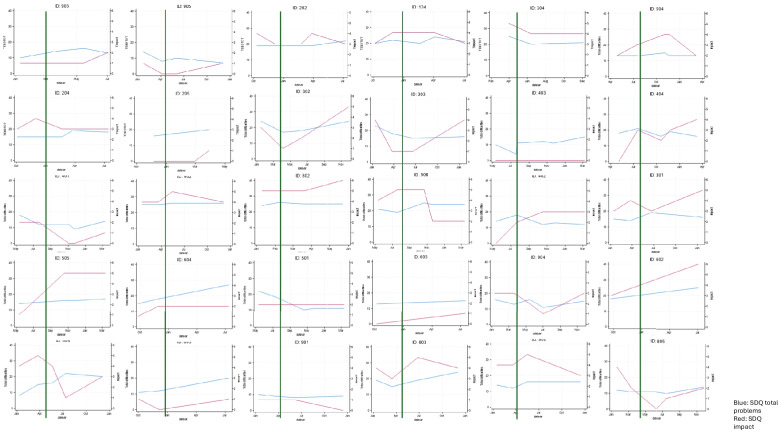
Graphical representations of child scores on Strengths and Difficulties Questionnaire over time Blue lines indicate SDQ total problem scores and red lines SDQ impact scores (teacher-report). The green horizontal line represents approximately when the intervention was introduced

Teachers reported that some children’s scores would have remained very stable (often where children were persistently absent and therefore less well known to the teacher), whereas others were constantly changing, likely related to the dynamic nature of ADHD. This was reflected in the individual data plotted over time (Figs. 3 and 4). Some asked whether questionnaires could be tailored to a child to remove any redundant questions, for example, when a child had never shown any externalised, aggressive or disruptive behaviour. Teachers believed that a personalised approach would decrease the time needed for completion.

Teachers believed that a maximum of one set of questions each half term would be most appropriate. They discussed how they experienced more challenging behaviour from all children at the start and end of a school term, indicating that future questionnaires should be consistent in terms of timing across the school term. Further detail on questionnaire feedback is shown in Supplementary Material S7.

Parents that were interviewed reported enjoying completing the study measures, and that the language and content were not difficult to digest. They liked the ways in which the study team communicated with them (a combination of phone, email, text and in-person visits at home or school), and how researchers tailored this to individual needs and preferences. Reminders were considered useful and not intrusive. Some parents, whose children did not have a diagnosis of ADHD, considered some of the questions in the resource use questionnaire to be unnecessary.

It was unanimously reported that children enjoyed researchers visiting in school to complete child-report measures. Teachers reported that this made the child “*feel heard*”. Children received a sticker for each questionnaire they completed, and they reported that this made them feel proud. It was implied by adults that a questionnaire measuring sense of belonging may be useful for children.

#### Perceived improvements on classroom functioning, ADHD symptoms and mental health

Mixed models assessing change in SWAN, SiSS-PB and SDQ total difficulties and impact scores (all teacher-reported) showed no evidence of change across the whole dataset, or for the 21 children for whom teachers reported using at least two intervention components. However, the qualitative data indicated that teachers and parents perceived positive impacts, relating to the items measured across the SDQ total difficulties score: peer relationship problems, hyperactivity/inattention, emotional problems and conduct problems. Overall, 22 teachers mentioned improvements that would map on to the SDQ, often listing two or three specific areas per child. Similarly, 18 parents reported perceived improvements at school that align to the SDQ questions. The SDQ was perceived as easy and meaningful for teachers to complete, and as such, we propose a definitive RCT use this as a primary outcome measure, with school-based impairment and ADHD symptoms as secondary outcomes. In an aligned study, the observational measure used (SCOPE classroom observation checklist) was found not to be valid, and as such, alternate measures such as the Behaviour Observation of Students in Schools [40] may be more appropriate [32]. A further consideration is that many of the perceived improvements related directly to the proximal goal that teachers reported working on (often selected via the behaviour web), so use of goal-based outcome measures could also be considered [41].

## Discussion

We report on the feasibility and acceptability of a novel individualised behaviour change intervention for teachers to support students with traits of ADHD age 5–10 years old in mainstream school settings. The co-designed intervention was positively received by school staff, families and children and perceived to be needed, useful and potentially beneficial. With some modifications made to the initial design of the Flex toolkit, schools perceived it to be highly acceptable to implement in mainstream primary schools.

Implementing Flex as an unguided intervention, a model we initially selected as it would result in the lowest possible intervention delivery cost, is not feasible for school staff. However, participants clearly articulated models of delivery that they perceived would improve implementation and fidelity, notably the support of trained “coaches” to liaise with teachers and dedicated time to select components from the toolkit to implement. Applying the Consolidated Framework for Implementation Research (CFIR) to understand the nature of the barriers, it is likely that these operate at multiple levels [42]. Staff time and capacity to deliver the intervention are likely contingent on *outer setting financing* constraints, with schools operating within limited budgets. The key implementation barrier seems to be related to the inner (school) setting, with a *lack of available resources* in terms of staff capacity. Interestingly, the solution proposed by staff was to increase *relative priority* by engaging external expertise to legitimise time to plan implementation, through use of *implementation facilitators* taking the form of coaches, who would also improve access to knowledge and information through expert guidance. However, there are other implementation domains that could be leveraged to overcome these barriers, for example, improving the *compatibility* of the toolkit with existing systems and processes, or altering the *work infrastructure* to encompass responsibilities for delivering the toolkit into staff roles. Systematically investigating any further barriers in line with the CFIR in a subsequent evaluation is important [42].

School staff have suggested that a light-touch coach could overcome these challenges, and are used to working within the constraints of the system without the ability to exercise systemic change. However, for widespread effective implementation, it is likely that systemic shifts will be needed. The recent Government White Paper on SEND lists reforms that will improve implementation of Flex, including provision of “Experts in Hand”, increased workforce and capacity of allied professionals including Educational Psychologists, who can be drawn in by schools as needed [43]. This will be supplemented by a commitment to increase the teaching workforce by 6500 posts in the Plan for Change that should also result in increased capacity within schools [44]. These systemic changes will however be slow to come to fruition and overcoming implementation barriers to Flex in the shorter term is still of utility. Furthermore, shifts in the Office for Standards in Education, Children’s Services and Skills (Ofsted; the regulatory body for education providers in the UK) framework to increase focus on inclusion of children with additional needs, should also leverage systemic shifts in favour of implementation of interventions such as Flex [45].

Coach-supported implementation will increase the cost of the intervention; however, it will be important in determining whether it is efficacious or effective and cost-effective for outcomes of importance. As suggested by participants, assessing an intervention model with external expert coaches such as educational psychologists (EPs) presents a logical next step. Following further consultation with educators and experts, a model that is considered feasible and likely sufficient would be EPs training teachers and the SENCo to use the toolkit over a half-day, or series of 2–3 after-school training sessions. The EP coach would then support implementation in the form of fortnightly 15-min teacher consultations to ensure adherence to the structure of the Flex process and to support teachers to problem-solve barriers. In turn, EPs would attend a full-day training on use of Flex with the intervention developer, and have access to drop-in sessions with the intervention team throughout their coaching role. This process and how it relates to overcoming implementation barriers require testing in an empirical study, ideally before progressing to a full-scale RCT. This proposed coaching process would combine expertise in ADHD (held by EPs, as SENCos often feel they are not sufficiently expert) with expectations on the school that ensures teachers will be given time to implement the toolkit (which may not happen if coaching is done within the existing staff body). If effectiveness is evidenced with EP coaches, then further research can determine the minimal additional needed input, or generalisability to other potential delivery agents that will incur lower costs (such as trained SENCos) to maintain effectiveness. 

In terms of resource implications, preliminary calculations suggest that if Flex were to be delivered to 17 children within a school by eight teachers with EP coaches, the intervention cost will be around £211/child in current prices. Other options that may overcome implementation barriers include Flex being adopted within Multi-Academy Trusts, with mandated use by teachers being subject to within-trust monitoring and evaluation systems. Capturing accurate data on fidelity to the intervention was challenging. These related mainly to the cognitive load that teachers operate under, and struggle to recall after several weeks what components they had interacted with. Embedding weekly fidelity measures would capture more accurate data on implementation. These data could have been captured through the login-interface of the toolkit in the feasibility study. However, teachers tended not to log in to the toolkit once they became familiar with navigation. This meant that we were unable to track fidelity within the platform to the degree that we had anticipated. In an RCT, it will be important to require log-ins; this will ensure that both user data are tracked and schools assigned to the control arm do not have access to the same intervention components.

Despite these challenges, the data captured indicated that most participating teachers engaged with at least some components of the toolkit, with most using at least two. This suggests that the toolkit materials themselves were feasible and acceptable to staff, with minimal changes suggested beyond the first four schools. This is a promising signal, given that evidence shows that behavioural interventions in school for children with classroom-based ADHD impairment are strongly supported. A recent review from experts report findings that parallel the content of the toolkit (clear goal setting, positive consequence-based strategies, frequent feedback), as well as our conclusion as to the change needed, with educators benefiting from ongoing coaching and monitoring [16].

While participating families reported that the research team’s communication was useful and positive, there remained wide variation and barriers to home-school communication from both parent and teacher perspectives. This meant that one of the best-evidenced components, the daily report card (DRC) [46], was not feasible. Although DRCs are well evidenced in US literature, there are no studies in the UK of which we are aware. It may be that within the UK contemporary school context DRCs are not implementable. However, the limitation of excluding the DRC in a future evaluation may dilute the potential efficacy of Flex. Exploration of other methods to improve home-school collaboration, modelled on the DRC format, may be necessary to generate a component that addresses this aspect of the logic model, as home-school relationships have been shown to be a major barrier for children with ADHD [47], and tensions between school and home may exacerbate challenges for children. This could be further explored through consultation with school staff as to feasible models of communication and the resources that would be required to implement and sustain these, building on the findings of this feasibility study, so that a modified and resourced version of the DRC could be built into a pilot trial to assess whether it can be integrated. An alternative option is to conduct an evaluation of DRC as a stand-alone intervention as additional burden was one reason for it not being feasible within the context of Flex. Without additional components, the DRC alone may be a manageable intervention for school staff.

One other component that families reported to be critical in the intervention co-design process was the careful and planned setting of individualised goals and targets for the study child, with the original prototype calling for two meetings between school and home and a functional behaviour analysis. The reason that this was infeasible was the mismatch between the expectations of resource needed (i.e., 1 h per child) and the resource available within schools per individual. Indeed, the resource and staffing constraints that schools and staff were under should not be underestimated; qualitative data showed that many of the study teachers worked at least 12 h a day to meet their usual responsibilities. This aligns with national data showing that the mean teacher working hours per week is 52.4 [48]. The level of priority that individual children can be given by a class teacher was considered to be a challenge by our study teachers, complicated both by other children whose needs were seen as more prominent, and the need for equity across the children in the whole class. Teachers demonstrated an aptitude for efficiency, and engaged with the toolkit in the most efficient manner possible for them, using the drop-down menus as proxies for behavioural goals. Ensuring that goals are formalised is likely to be important in terms of the logic model of change. Integrating this with existing school-level structures for goal setting may be the most appropriate method. Alternately, the behaviour web feature of the prototype could be reconfigured to allow teachers to select a goal from this list and personalise or add this to their student’s toolkit.

The research design was feasible and acceptable to school staff and families. This is highly encouraging, given that the data collection schedule for this case series study was intensive, with teachers providing data every 2 to 3 weeks for a year. Recommendations were made regarding the length and frequency of some measures. However, completion rates overall were good across all participants. Ensuring that a trial proactively plans for inclusion of families from varied backgrounds will be critical, as many of the reflections indicated that relationships built by the research team meant that reminders and offers to complete measures in person were perceived as helpful. As ADHD is highly heritable, families of children in the study were likely to have high levels of ADHD traits themselves, and so reminders and in-person visits were critical in maintaining engagement.

We therefore deem a future definitive trial to be feasible in terms of research design, with these considerations addressed. The challenge of blinded outcome measures remains a notable problem within the field of non-pharmacological school-based mental health treatments [49]. Teachers are often the only adult who knows the child well enough to report in detail on symptoms and impairment. Yet as they receive the intervention, they will not be blinded to treatment allocation. Use of two approaches, a blinded observation of classroom behaviour, such as the Behavior Observation of Students in Schools (BOSS) in conjunction with a “probably blinded” measure may be the best approach. Parents often reported no detailed knowledge of the use of the toolkit in school. Given the challenges of integrating a daily report card into the intervention, one option would be to test the efficacy of the toolkit with no explicit school-home component, and use parent-reported impairment at school on a measure such as the Weiss Functional Impairment Scale [50] as the probably blinded outcome.

The measures that were used were considered to be appropriate by adults and children. These were chosen through the co-design process. However, the SWAN and SISS-PB that were potential co-primary outcomes for a trial showed wide variation across individuals in the study and were not considered to be the most appropriate for capturing change that was perceived and reported in the qualitative data. Indeed, the theory of change posits that school-based impairment will be the most proximal change; however, there are no validated measures of this specific to populations with ADHD of which we are aware. We did however find clear evidence across the qualitative data that both teachers and parents perceived positive impacts of the toolkit on behaviour at school, indicating proof of concept. The behaviours perceived to improve varied, as would be expected given the heterogeneous nature of ADHD, but many of these are captured on dimensional mental health measures such as the Strengths and Difficulties Questionnaire. The SDQ total difficulties may be an appropriate primary outcome in an RCT; however, goal-based outcome measures should also be explored further. The SDQ was perceived as easy and quick to complete by teachers, and the qualitative data on perceived change from both school and home indicated that improvement in school-based mental health impairment was the most important consideration for both school staff and families; this can be captured using the same instrument. Utilising this as a continuous measure would evidence change for an individual child in areas that they face impairment in, across the breadth of conduct, emotional, peer problems and hyperactivity or inattention, and has the advantages of mapping onto health economic analyses [51] and being well-utilised in evaluations of school-based interventions [52]. A large-scale trial suitably powered to assess change in relation to the logic model, and employing methods such as qualitative comparative analysis or realist evaluation [53, 54] that demonstrates which combinations of components are associated with efficacy, would enable assessment of specific mechanisms that result in positive outcomes.

We captured data on adverse events from all study participants, and in no cases was the intervention perceived to cause adverse impact. One concern the planning group had was that children may feel singled out by the intervention, leading to upset and isolation. However, the inclusion of both individual and group/class-level strategies meant that teachers selected components they thought that the child would respond to, and there was evidence to suggest that children enjoyed participating. Interestingly, both teachers and children varied in their preference for whole-class or individual strategies, suggesting that the inclusion of a diverse range of strategies was appropriate.

Although we captured detailed quantitative data, the study was not designed or powered to detect any impact of the intervention on these measures, rather to assess their suitability for a future evaluation. It is therefore unsurprising that we did not observe change in these outcomes, and additional analyses that were proposed in the protocol, comparing between those who did and did not use the intervention with fidelity, were not conducted due to the challenges of robust data capture on components delivered. However, there are other reasons that change on quantitative measures may not have been observed, including measurement insensitivity to proximal, individualised goals. This is highly possible, given that the qualitative data captured perceived improvements as being very proximally linked to individual goal and strategies chosen that may only have been quantitatively captured by one question on the SWAN or SDQ. Another potential explanation is misalignment between intervention mechanisms and the selected outcome constructs. Indeed, the most proximal outcome specified in the theory of change is reduced impairment, and improved functioning in specific areas of difficulty. More distal outcomes relate to mental health improvement, and ADHD symptom change is not a proximal goal of the intervention, taking the stance that these traits are inherent to the child and not amenable to sustained change through environmental interventions.

There may also be contextual volatility in ADHD behaviours across school terms, although due to the staggered nature of the case series study (with children having study starts in September, January and April), this would have been averaged out across participants. There are no studies of which we are aware that measure symptom variability in ADHD across the school year. In addition, there may have been social desirability and/or expectancy effects operating in the qualitative interviews [55, 56]. Teachers were aware of the aims of the study. The topic guide did imply that some engagement had been had with Flex, and asked directly about perceived behaviour change (although this did cover both improvement and negative impacts) and so demand characteristics such as this may have influenced perceptions of improvements for individual children. Measurement of treatment expectancy in future evaluations and triangulation of data from different sources will be important.

Regarding fidelity, as many teachers ceased to log in to the digital platform once they had selected a strategy, and struggled to recall their activities with precision over the 6-weeks covered by each process evaluation, this was not tracked in the way originally intended. Systematic measurement of dose delivered and dose received is essential to draw any inference as to reasons for the absence of quantitative change, although this would still not overcome that this study was not powered to detect or understand quantitative change. A subsequent evaluation will need to ensure fidelity is measured regularly and robustly to ascertain which components are associated with efficacy. To ensure this, Flex will only be available via login, and we will employ fidelity monitoring procedures, encompassing adoption, intensity and quality of implementation. This will require capturing data on content, process, quality and dose by adapting those used in studies of the Collaborative Life Skills intervention [57]. This will include brief fortnightly “pulse” surveys to teachers with a checklist of components to indicate use and frequency of use, website analytics, recording teacher-coach sessions and training to code against adherence to the manual, and observations of classroom practice to capture evidence of use of toolkit strategies in practice, resources printed and evident in teaching and planning materials. The only teacher burden in this process is the pulse surveys, and as teachers were able to complete repeated measures during the feasibility study with high compliance we are confident that this will not unduly increase burden. Nonetheless, many of the measures chosen were considered to hold face validity for participants, with many of the items of the SDQ relating to qualitative data on perceived improvement. A second option could be use of goal-based outcome measures, as these will link proximally to the individualised targets selected for each child [41].

### Strengths and limitations

The iterative case study approach has both strengths and limitations. In terms of meeting the study aims, refining the intervention across participating schools meant that acceptability and perceived usefulness was high by the end of the study. However, it also meant that children in each school received different versions of the intervention, and so statistical analyses of data or comparisons have limited utility. Indeed, the study was not powered to detect change on quantitative measures and so this does not give a clear indication of the outcome that may be most responsive to the intervention.

In addition, we began from a starting point of the lowest-cost intervention model possible, with very limited input for schools beyond initial orientation to the toolkit. Given the significant implementation challenges faced by staff, this means that detecting robust change was unlikely. We recommend that a RCT has a built-in pilot stage in order to ascertain whether the primary outcome measure indicates promise. Evidencing efficacy or effectiveness of school-based interventions is often challenging [58, 59], and supplementing standardised measures with high-level data on perceived change may be useful.

## Conclusions

This study reports that a novel non-pharmacological school-based intervention for children age 5–10 with high levels of traits of ADHD is acceptable for school staff and families, and our findings suggest that it would be feasible to implement to high fidelity with some extra guidance. We have demonstrated that conducting a high-quality evaluation of the intervention will be feasible, and shown tentative proof of concept of the intervention to impact on outcomes relevant for children with ADHD. Large-scale evaluation of the intervention with a built-in pilot phase to ascertain the most appropriate outcome measures is feasible.

## Supplementary Information

Supplementary Material 1: S1 to S5.

## Data Availability

The datasets generated and/or analysed during the current study are not publicly available due to variation in permission from participants for data sharing, but data with appropriate permissions are available from the corresponding author on reasonable request.
