# Supplementary material for: Assessing the feasibility and acceptability of a school-based non-pharmacological intervention for ADHD: the Flex toolkit
Source: Pilot Feasibility Stud. 2026 Jul 22;12:106. doi: 10.1186/s40814-026-01879-7 (PMC13425861; doi:10.1186/s40814-026-01879-7)
Supplement: Supplementary file 1 — Supplementary Material 1: S1 to S5. [file 40814_2026_1879_MOESM1_ESM.docx]

# Supplementary material

## S1. Flex toolkit description

Flex is a school-based intervention for ADHD and associated traits co-designed using the Implementation Mapping approach (26), a method based on integration of existing evidence with theory and lived experience, and rooted in principles of behaviour change. Flex has been co-designed with a planning group of children with ADHD, families, school staff and experts (e.g. educational psychologists) to meet the needs of mainstream UK primary schools. A needs analysis was conducted by interviewing people with ADHD about their experiences of school, and what could be improved, as well as experiences and perceptions of school staff and families. Logic models were created of the worst-case (badly-supported ADHD in school), best-case (well-supported ADHD) and the process of change from worst to best case scenario. A separate publication will provide further detail on the toolkit co-creation process (Russell et al., 2025, *in preparation).*

This logic model of change was then used to identify the key target behaviours for the relevant agents within the school system, including senior leadership, teachers, support staff, peers and students with ADHD. Working with the planning group, targets behaviours were prioritised. Each of the target behaviours was broken down into the smallest behavioural steps, or ‘performance objectives’ that each agent would need to follow to attain this target. Figure 1 shows the theory of change. A comprehensive review of reviews of studies targeting the outcomes of interest was conducted in parallel to identify evidence-based components of interventions that were matched to these performance objectives. Strategies were designed that would enable a teacher to follow a series of steps, supported by resources for e.g. classroom guidelines, lesson plans and reflective templates, to implement each strategy. Supplementary Material S1 includes a range of screenshots from the toolkit.

## Flex toolkit website screenshots

## Landing page (senior leadership login version shown)


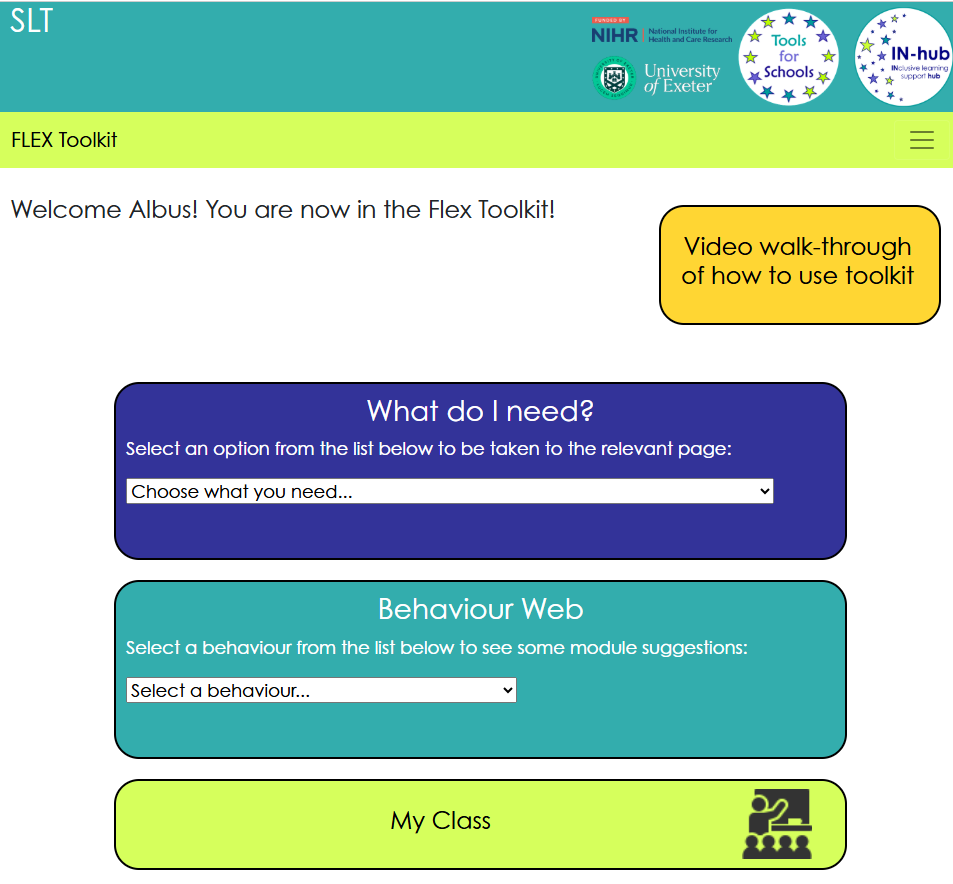


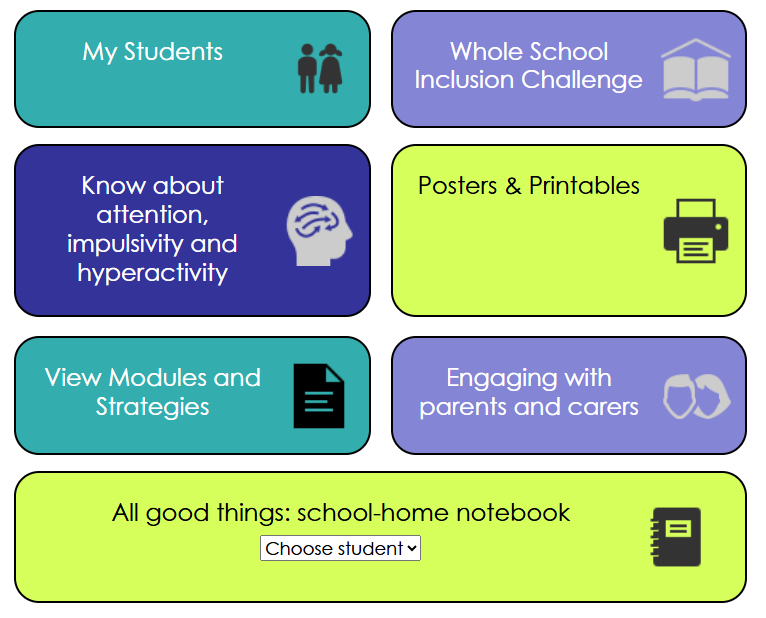


## Know ADHD page


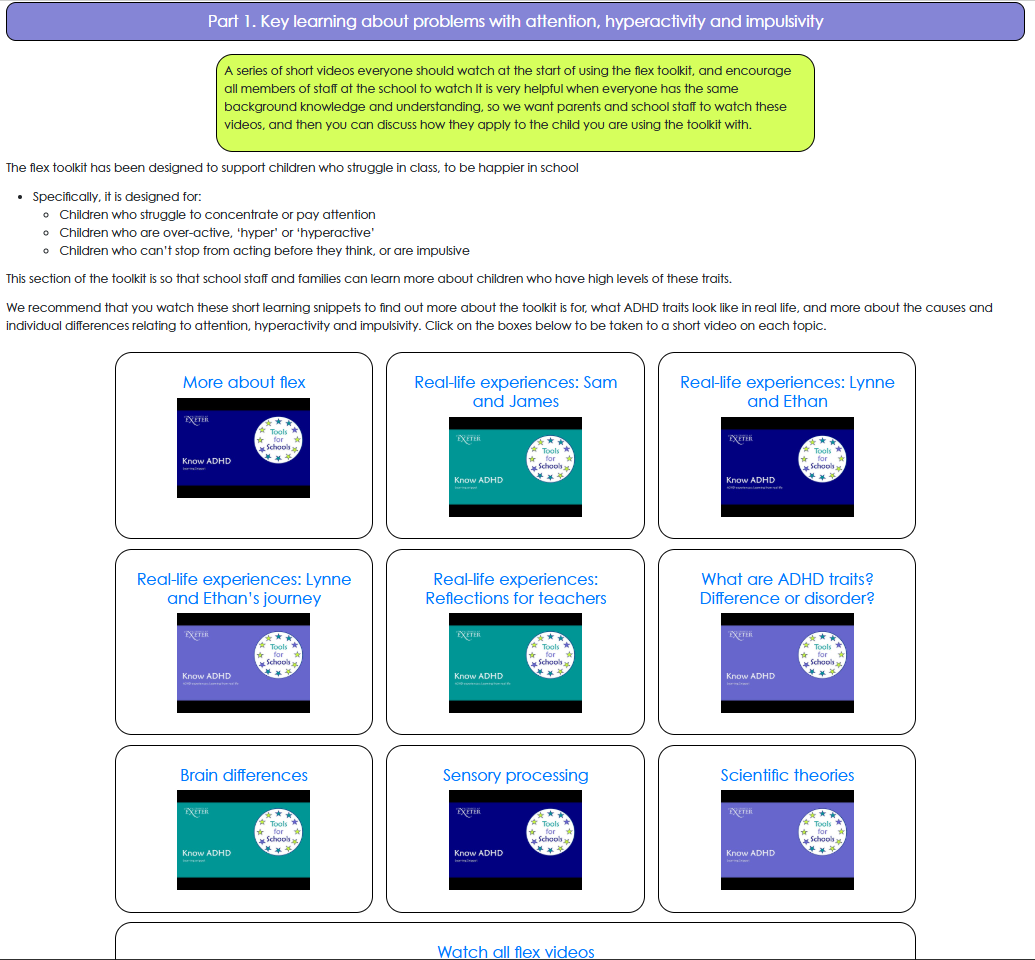


## Know Me page


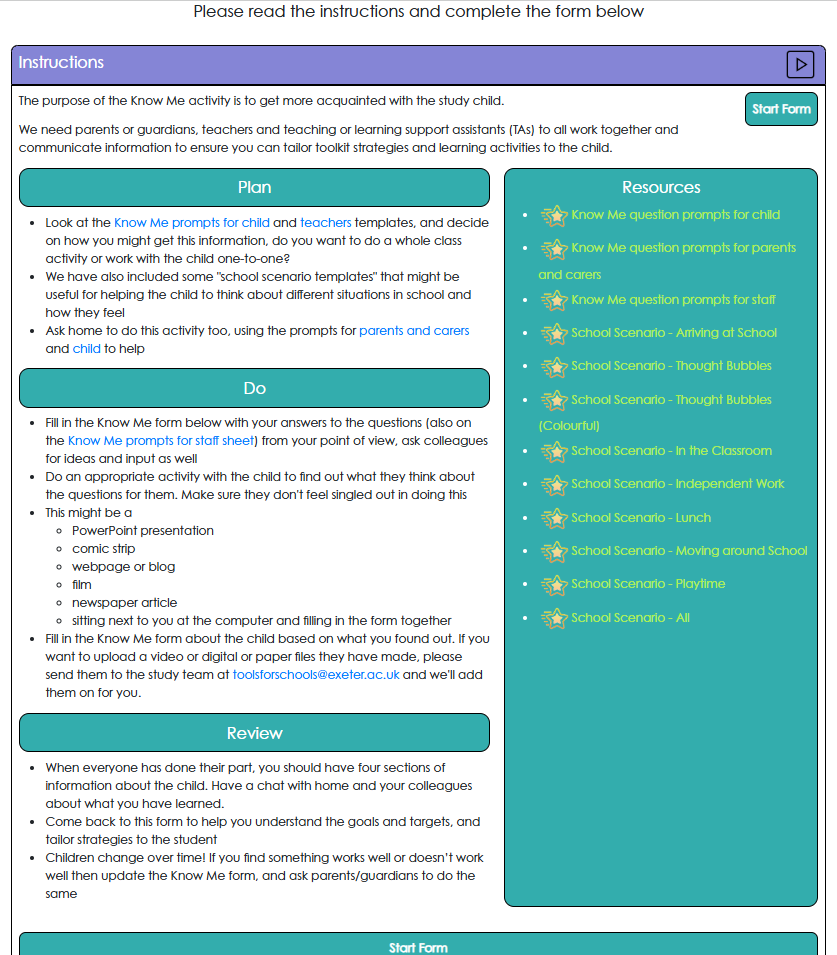


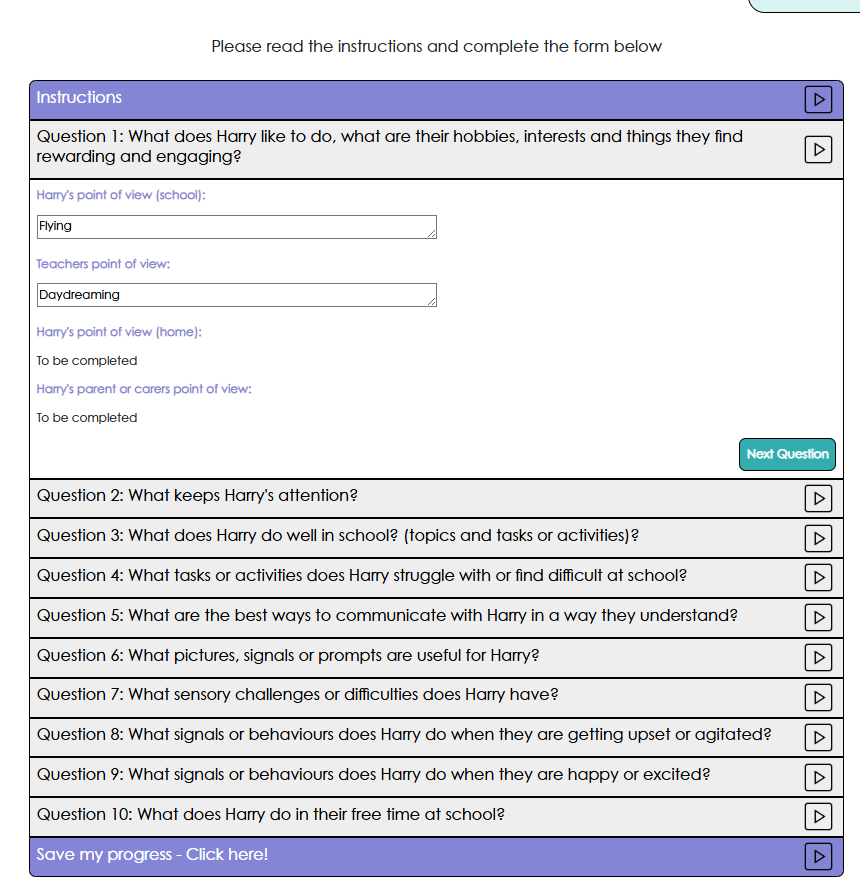


## Example printable prompts for home for Know Me activity


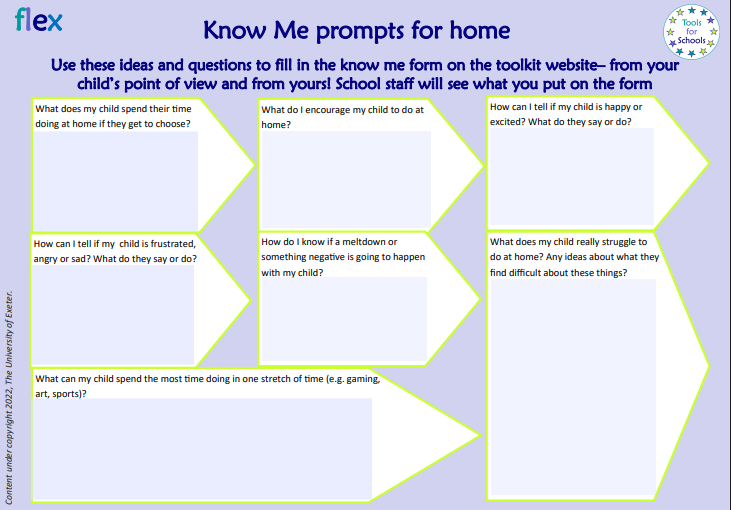


## Setting Goals and Targets page


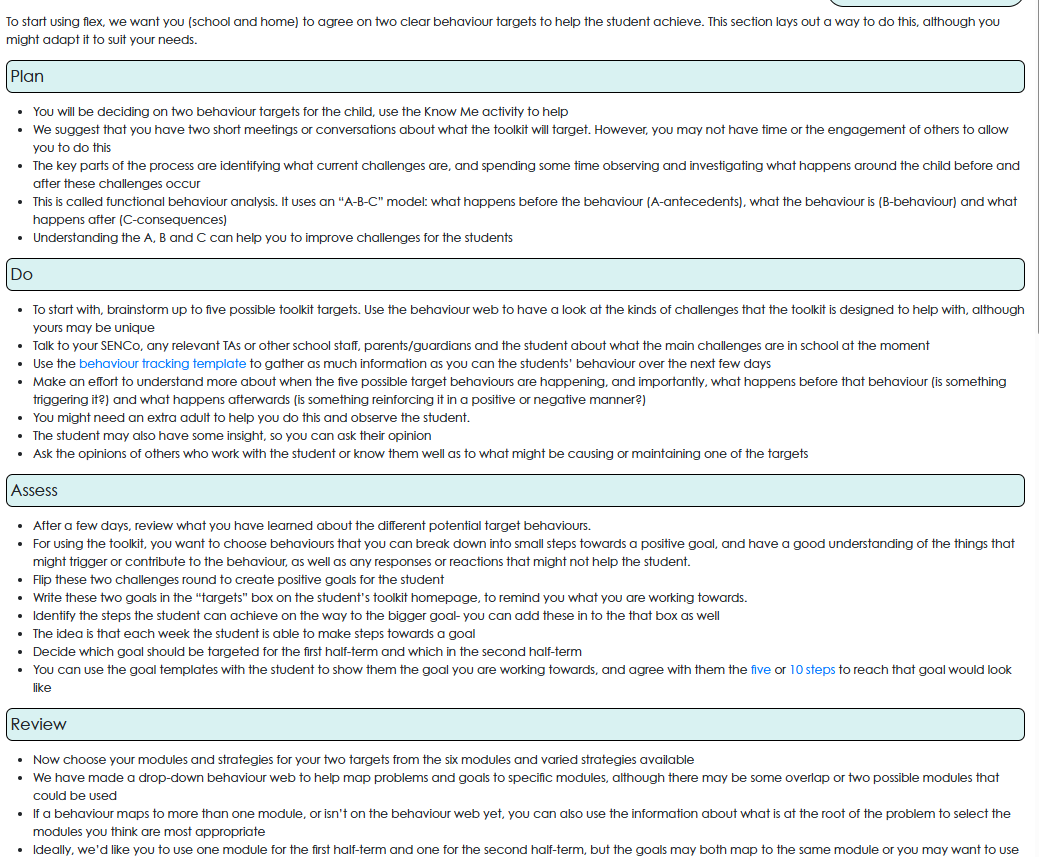


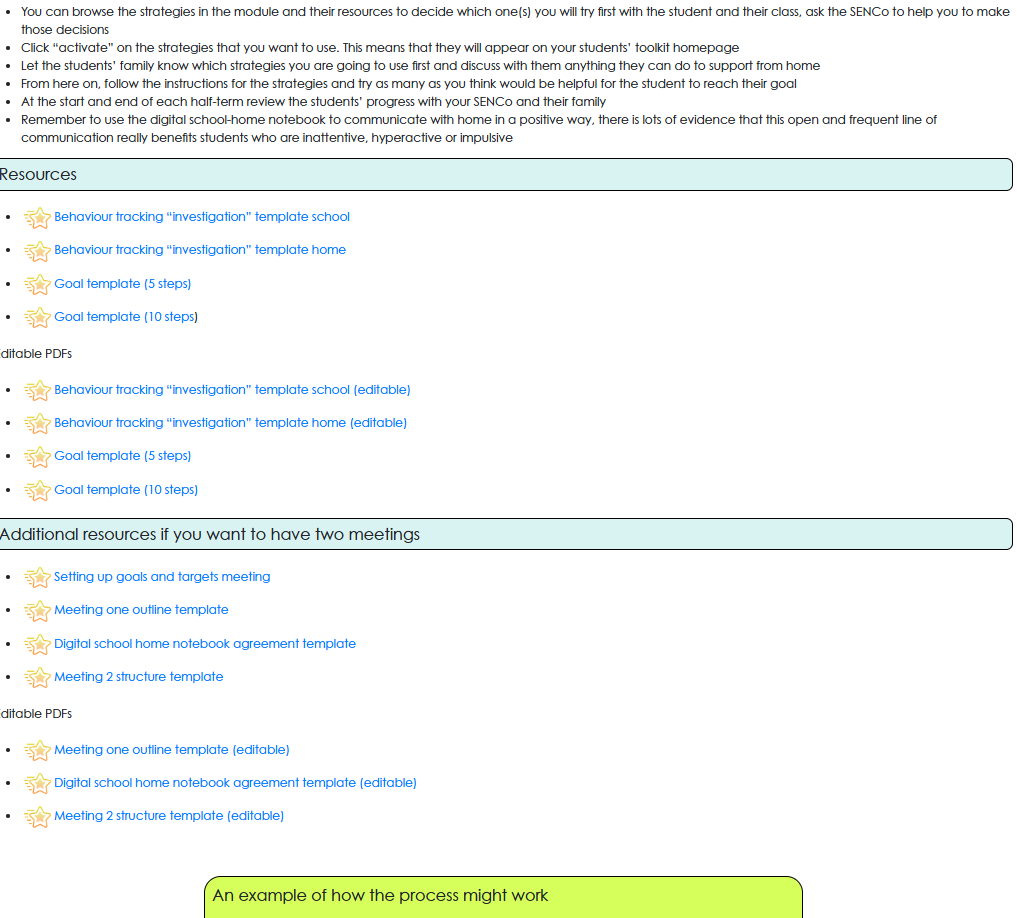


## Example meeting template for setting goals and targets


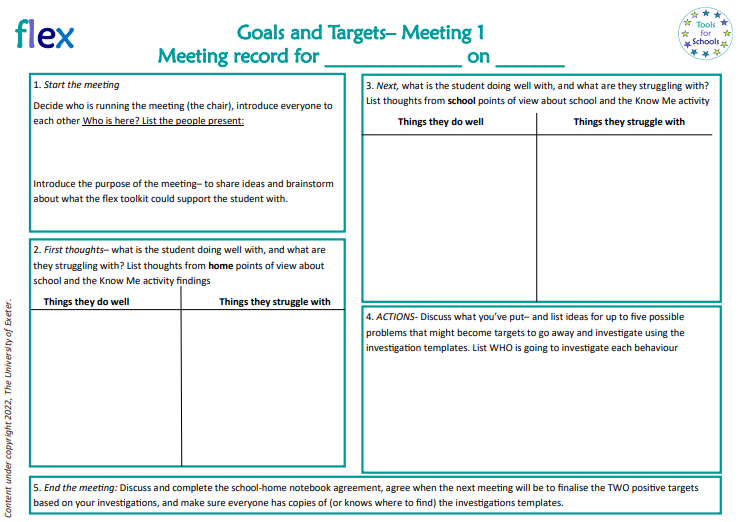


## Behaviour web
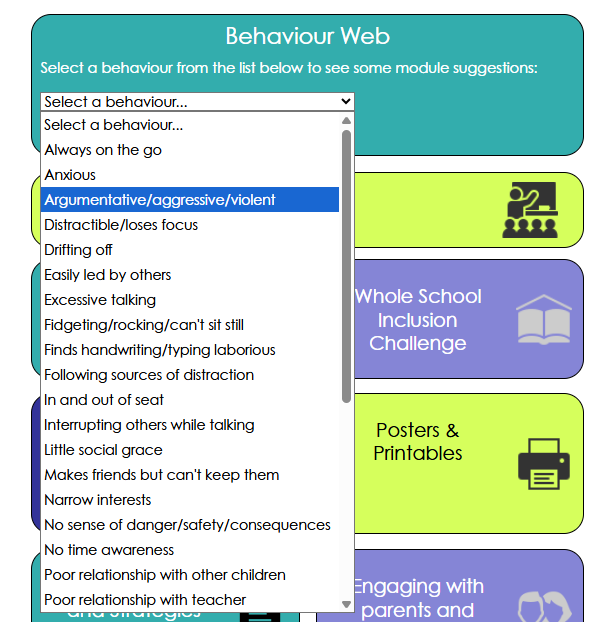


## Child toolkit page


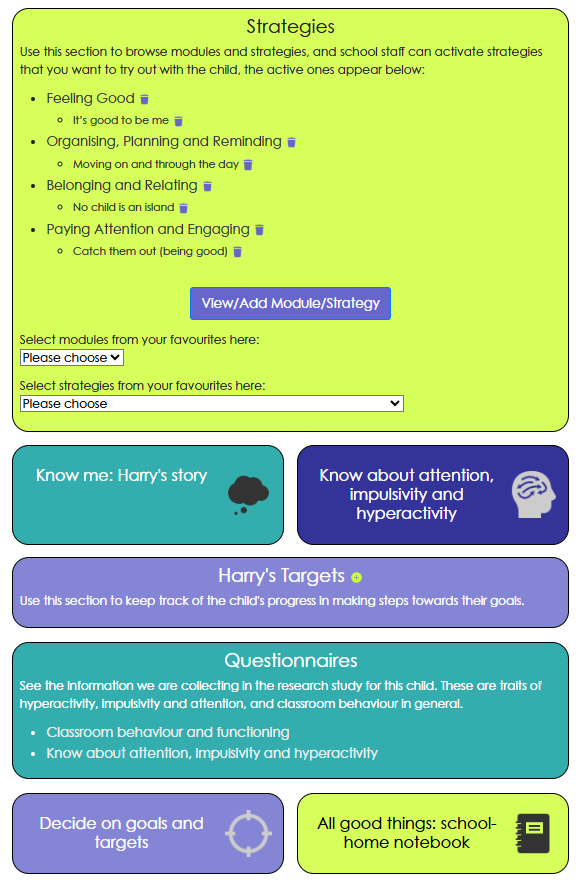


## Example module home-page with strategy short descriptions


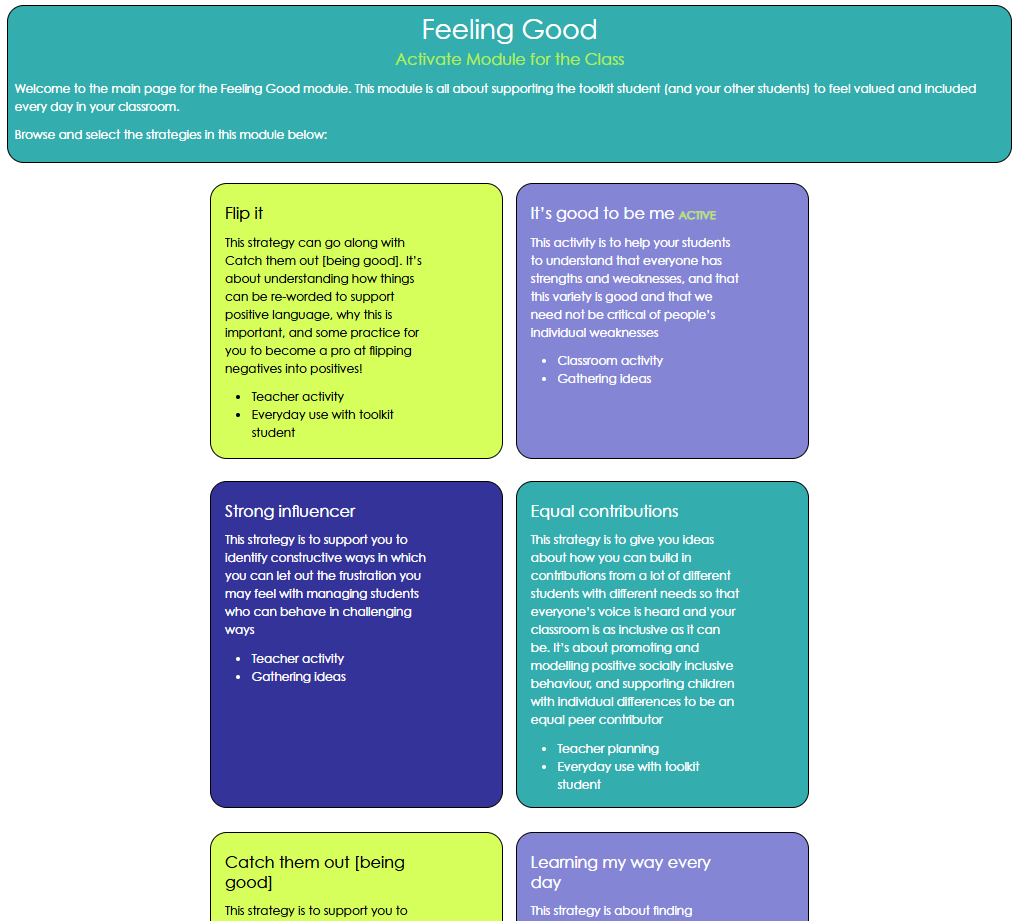


## Example strategy page


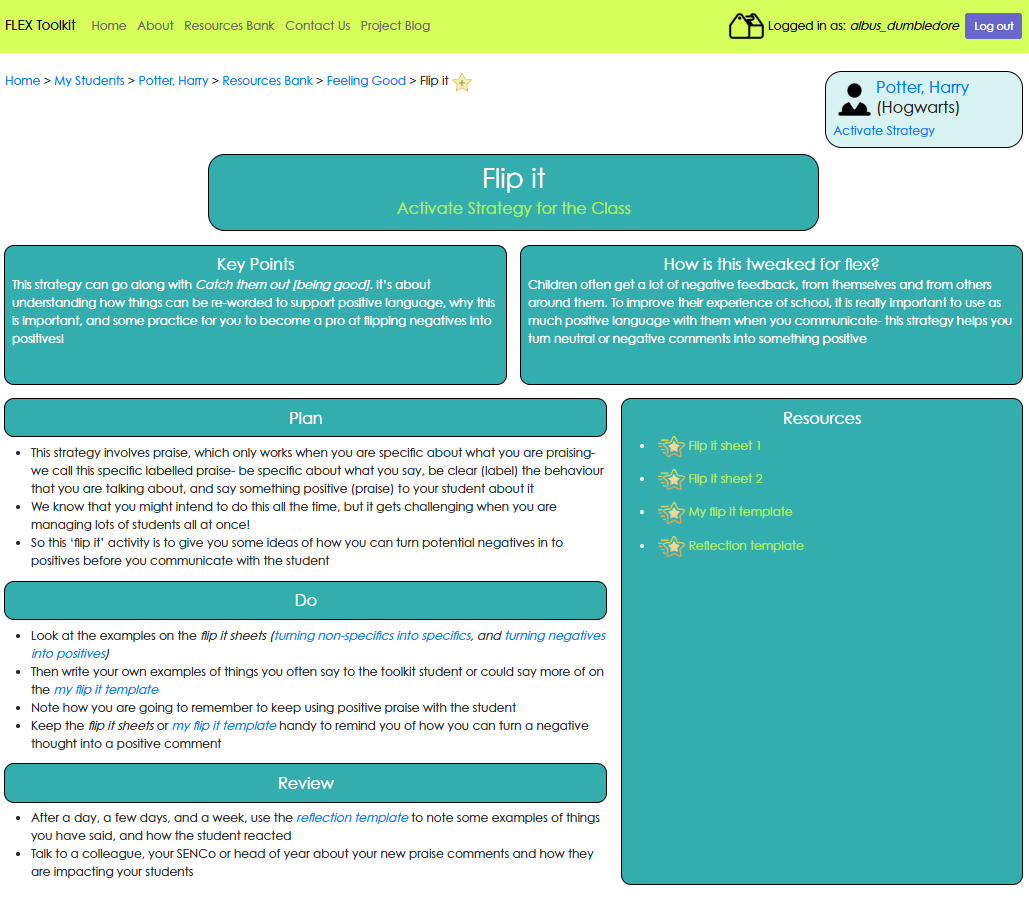


## Examples of PDF resources to accompany strategy delivery


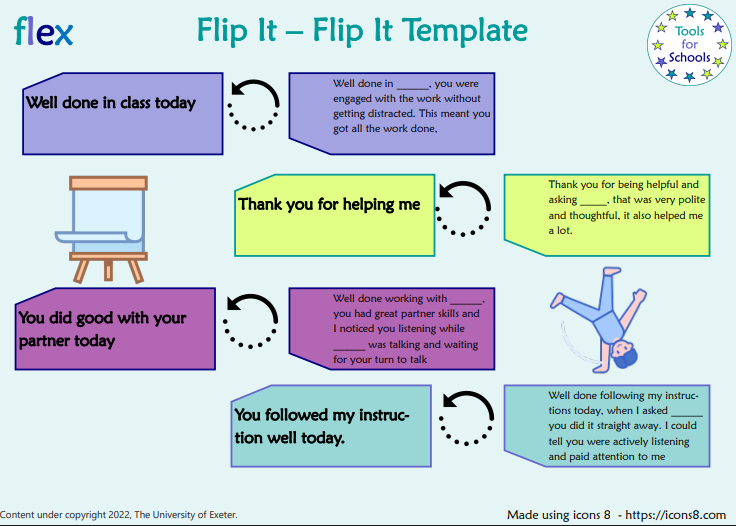


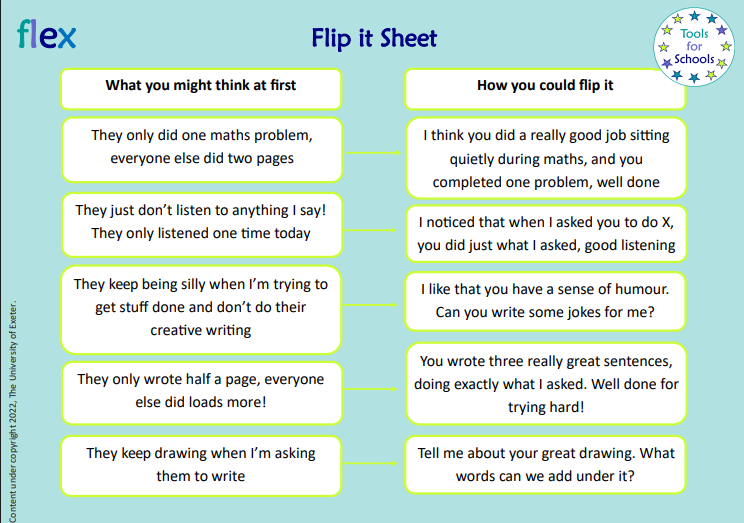


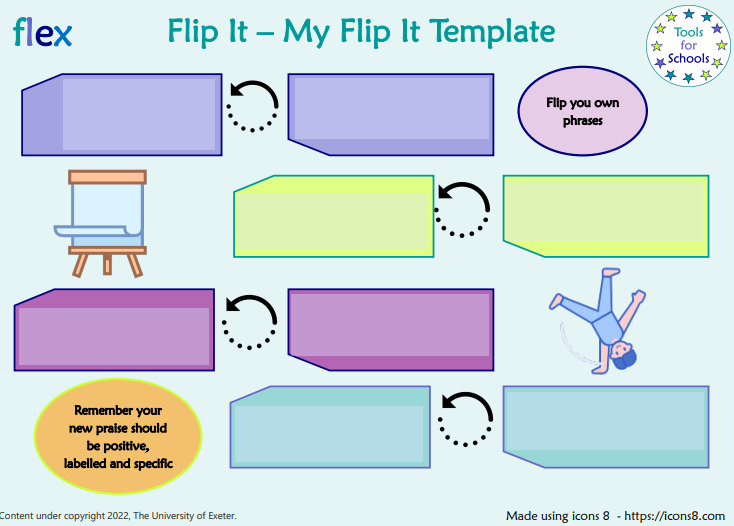


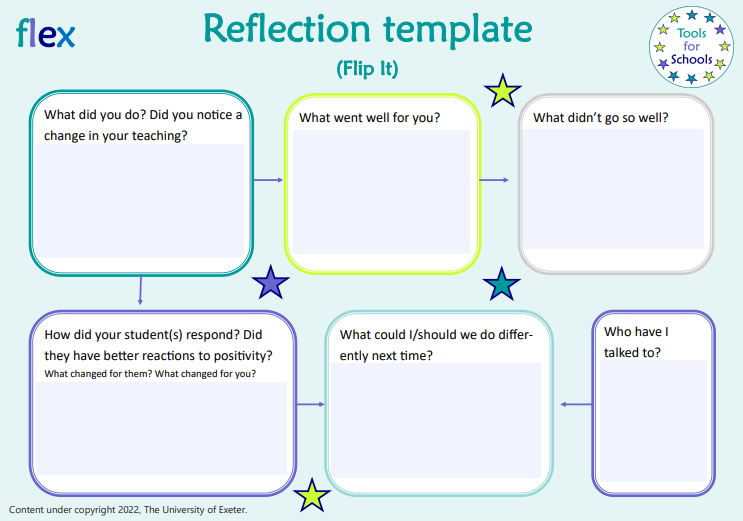


## S2. School sampling

Recruitment was promoted through discussion of the toolkit and study in professional network meetings (e.g. Special Educational Needs Coordinators (SENCo) inclusion forums), contacts of the local Educational Psychology teams, the Planning Group and other professional contacts and networks across the South West. Purposive sampling of interested schools ensured that a minimum of four schools were in areas of higher socioeconomic deprivation, based on the percentage of pupils eligible for free school meals being above the regional mean. Within each school, the headteacher and SENCo were required to consent.

## S3. Eligibility screening and recruitment

School SENCos worked with the research team to identify students with either a diagnosis of ADHD, or impairing traits. A screening questionnaire (the Strengths and Difficulties Questionnaire) was administered to parents and the class teacher if the child did not have an ADHD diagnosis. Eligibility required a “probable ADHD” status based on the SDQ algorithm (32): scoring ≥7 on the hyperactivity/inattention subscale by both informants, as well as being considered impaired by scoring ≥2 on the impact questions at either home or school. SENCos administered screening questionnaires prior to the research team having contact with the family. They were asked to consider nominating a range of children, in terms of age (5-10 years), sex (male/female), ethnicity (white/other) and family background. They also considered the classes that children were in to minimise burden on teaching staff (e.g. ideally one participating student per teacher).

Two to six children were recruited per school (schools ranged from 105 to 630 pupils). SENCos obtained verbal permission from families/guardians for the research team to contact them. A researcher visited the home to explain the study, obtain informed consent from the primary caregiver(s) (henceforth ‘parents’) for their and the child’s participation, as well as child assent where children were aware of the study (directed by caregiver’s wishes). In parallel, the study researchers also presented an overview of the project at a school all-staff meeting to familiarise the staff with the study. Once a child had been recruited, their class teacher (or teachers, in the case of job-sharing arrangements) were approached by the research team. Teachers provided informed consent after being given time to consider their participation. TAs were recruited, where they worked closely with a study child.

## S4. Resource use questionnaire


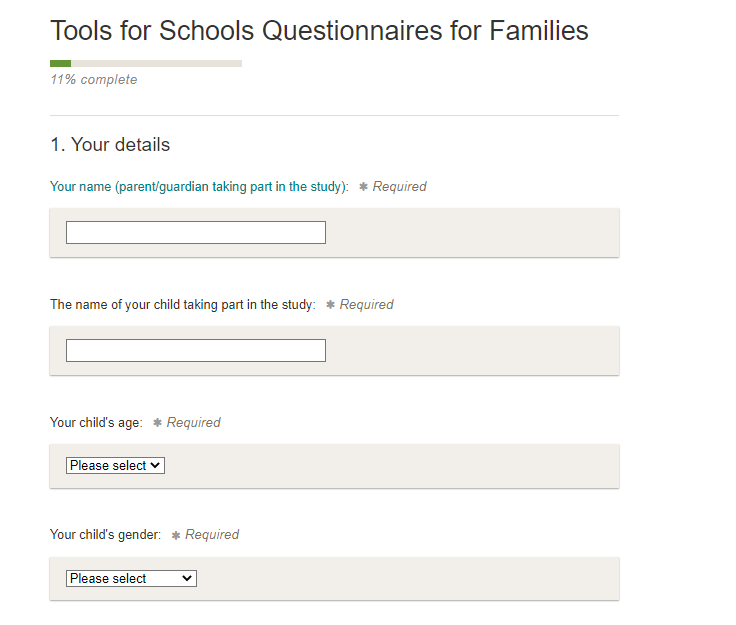


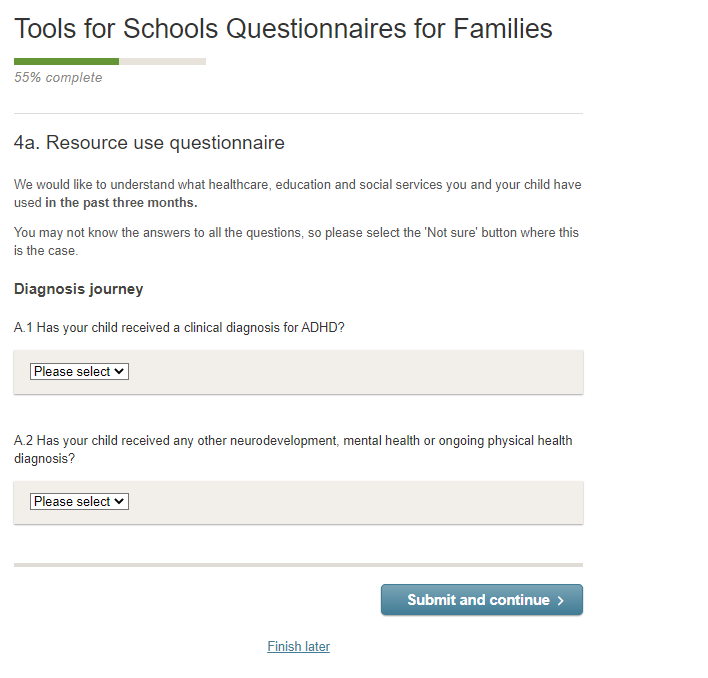


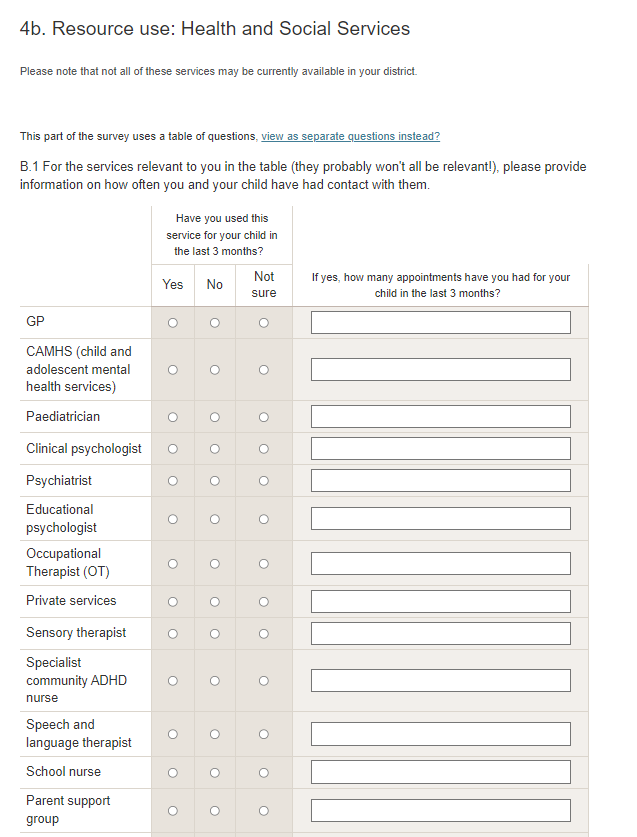


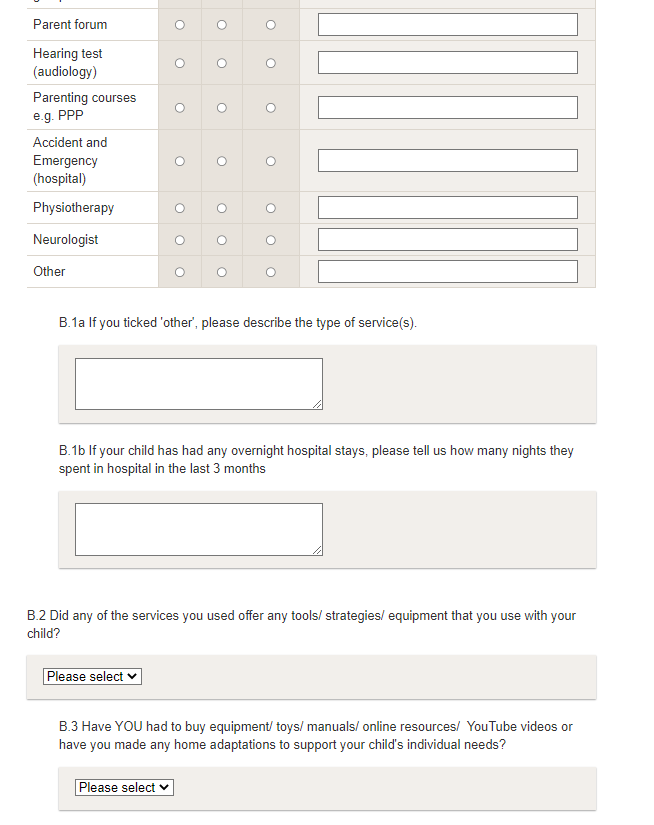


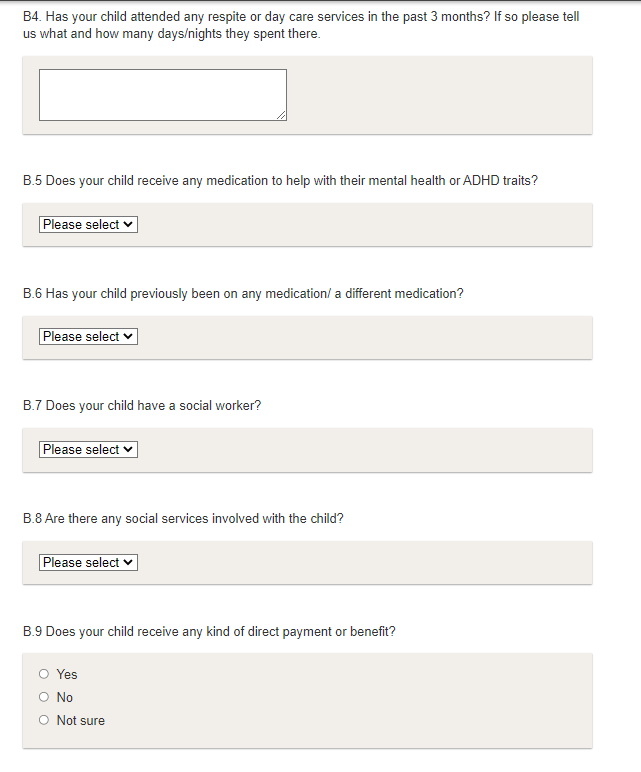


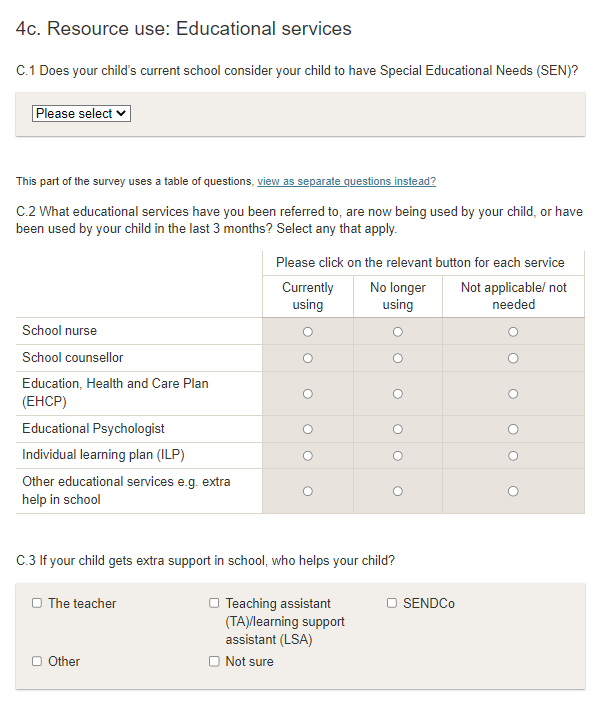


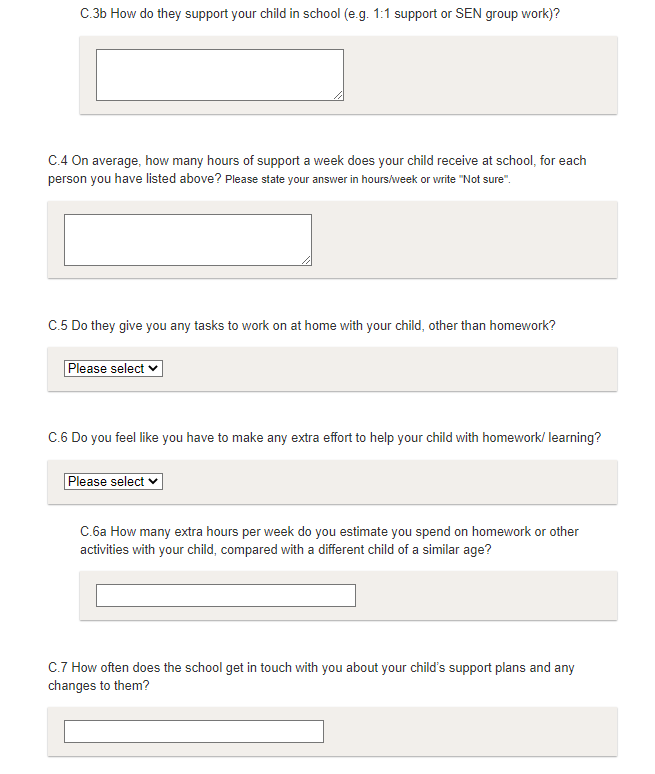


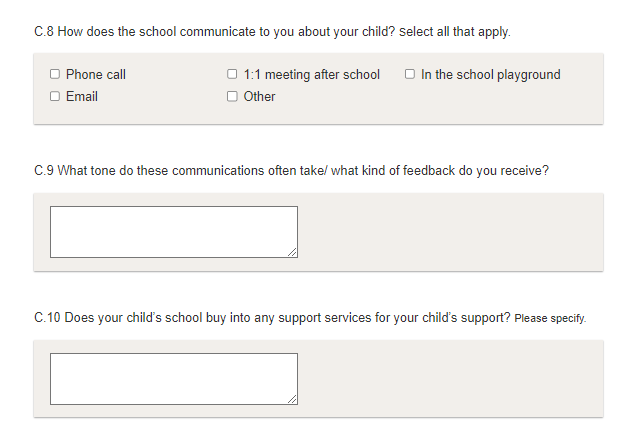


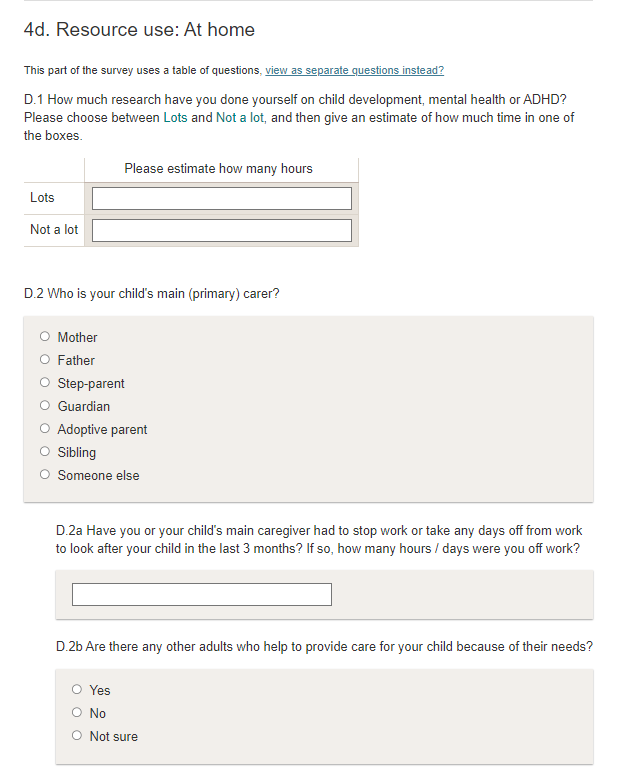


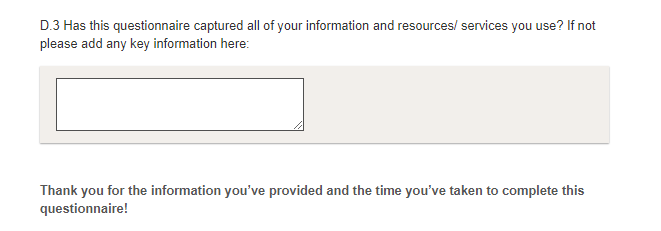


## S5. Supplementary Qualitative Findings

**Use of specific strategies and impacts on behaviour at school**

Teachers perceived improvements in their relationship with the child. This arose from the toolkit raising their awareness of the child in their class, relative to other pupils. This keeping of the child in mind meant that teachers were able to tailor their delivery and activities to engage them, informed by a deeper understanding of the child’s wish to meet the expectations and rules of the class, and their ability to consciously make positive choices. Teachers reported that a better understanding of the child, and increased awareness and understanding of ADHD, meant that they could make learning expectations appropriate to their level, and explicit to the child, which was motivating for the child in class.

Relating to this increased attention and focus on the child, teachers reported that they were better able to support them, using positive reinforcement with rewards informed by the child’s interests and becoming aware of when in the day to implement toolkit strategies to best support their needs. They perceived that children “*react well to praise*” and used this strategy often. They mentioned that strategies would work for specific contexts or times of day, and were explicitly aware of when these times were, allowing them to deploy strategies when they were most likely to have an impact. The “*carrots*” of positive reinforcement were considered very helpful in supporting the child to engage with work:

*“I was catching her out doing anything good, like sitting still for a couple of minutes, or putting her hand up, or engaging, or anything at all that she was doing that was positive I was praising her for it, so that was obviously really good, and I think also then that did happen with the rest of the class, the positivity spread a little bit, so that was really good. But yes, I do think it was beneficial for her”*

This active demonstration to the child that their teacher knows them and considers them as an individual in the class was important. Teachers considered that hearing the child’s voice was important and benefitted them and their relationship with the child:

*“I think he does react well to praise and I think because he’s very quiet it’s easy to forget that, unless you are deliberately making that decision and that effort.*”

Other behaviour changes reported by teachers included identifying and scaffolding opportunities for the child to self-regulate, interacting with the child in a different manner and so becoming less angry or negative in their attitude toward the child, and increasing the quality time they spend together. Teachers reporting a better understanding of what a child was thinking and feeling, and this led to an awareness in the child that their teacher cared about them as a person, potentially making children feel accepted and welcomed. This was also reflected from comments by parents that the teacher knows their child well.

With some strategies, teachers reported giving the child choice and increasing independence with the use of regulation strategies such as the Box of Tricks: “*…those are the ones he chose himself.* *So I’m thinking because he chose them, they’re working more so than if I’d chosen them*”. Teachers perceived that children really liked to have these choices, and in turn this supported both behavioural and emotional regulation. Some teachers reported benefits for their whole class: “*actually the impact it is for them that it has on the whole class and…attention and listening”.*

Teachers’ experiences with the toolkit were that there were often clear benefits for the child in classroom. This included an increased willingness to do academic work, often supported by the child being more able to stay physically within the classroom setting for longer periods of time. Teachers believed that when children understood expectations at an appropriate level, this was motivating for the child. Peer relationships were also reported to improve for some children, teachers reported that children were better able to make “*good choices*” and became “*less reactive*”, leading to fewer problems with friendships. They also reported strategies supported children to transition out of a negative mood, for example, if they did have problems with peer interactions, and children seeking adult support for help with peer interactions.

Other teachers reported the benefits of movement strategies for the child, mentioning multiple impacts, most commonly the idea that the child could “*reset*”. The impacts of this were that the child was more settled in class, did not lose track of where they were with their work, were able to “switch off”, especially when they had become hyperfocused on a task: *“I do think that helps her to switch off for a minute and kind of reset herself”*. The movement breaks meant that the child was able to pick the work back up where they left off as well, with teachers reporting fewer reminders and prompts being needed in class.

*“I introduced the movement mat at the start of the year, and that definitely helped [child’s] focus and engagement in the lessons, and I’ve found now he’s more independent with knowing when he needs to go and do something or he needs something to fiddle with, and he’ll do that quietly without making a scene”*

Movement, and other strategies, were reported to promote the child’s engagement with learning and academic subjects. This included increased organisation skills, better ability to focus, and children’s willingness to try with work. Benefits were reported for different children in reading, maths and the presentation and completeness of work. Many of these benefits were felt to stem from improved relationships, and increased use of movement or sensory breaks, leading to opportunities to regulate within the classroom.

**School-level considerations for trial design**

Selection of students. Teachers often questioned why the specific study children had been selected, and some mentioned that there were others within their class that they thought would be more appropriate for the toolkit and study. Giving teachers more agency in these choices would likely increase engagement and buy-in.

*School SEND culture.* Teachers reported wide variation in opinions of the active involvement of the SENCo in the study, ranging from “unsupportive” to fully engaged and proactive (for example by arranging meetings with parents, prompting staff to watch videos or use strategies). SENCos in some schools were part of the Senior Leadership Team, but not in others. Some worked part time or across multiple schools, and held multiple roles in addition to being the SENCo. This complexity sits within the wider SEND culture of the school; indeed a few teachers reported that their Ordinarily Available Inclusive provision was strong and there was limited potential for the toolkit to add benefit. Some schools considered all staff to be “very passionate about SEND”, whereas others were believed by parents to not “embrace” SEND . Parents also reflected on the challenges with SEND in schools, with some struggling to obtain an Education, Health and Care Plan (EHCP) for their child, others unsure if their child was on the SEND register, or being unclear how the funding attached to an EHCP for their child was deployed within the school to support them. Some children were sent home from school frequently, or parents were required to support their child within school, for example to supervise their child’s lunch. These parents believed that the school did not understand of their child’s needs, and further felt that schools needed to “help, not blame” their child with their behaviour. There was Parents perceived variation among teachers’ competency with SEND pupils, with one describing this as a “potluck” in terms of which teacher their child would be allocated the following year.

*Other staff.* Teachers believed that other staff in the school could benefit by being involved in the study, in particular teaching assistants (TAs). They suggested that the toolkit could be introduced to all TAs in one meeting, and then teachers would be able to utilise TA support in preparing resources and implementing strategies. Some suggested that the toolkit should be delivered to the whole school, or to allow schools to choose how many teachers they involved. They believed that buy-in at a whole school level would mean allocated time for teachers to use the intervention, perhaps within their CPD allocation. The benefits were that it would be of an equal priority for all staff rather than involving a select few, and the opportunity for impromptu conversations such as during lunch time and in the staff room. Teachers especially highlighted the value of hearing from their peers what is working well. Working at a whole-school level would also potentially enable use of SEND meeting time and staff meeting time to discuss the toolkit.

*Feedback on child/to teachers.* Teachers reported that they prioritise efficiency, and highlighted several points in the delivery process that could be streamlined. These included using child observations to feed into the toolkit, or using the research data to direct to strategies to select. Teachers also reported wanting feedback on the child’s progress. Interestingly no teachers reported looking at the study data that were presented within the toolkit (scores over time on the SWAN and SISS-PB scale) but instead were more curious about the purpose and findings of the behaviour observations conducted in the classroom each half term. Teachers report being accustomed to receiving feedback if a child is observed, and some were unclear about the purpose of the observations.

*Communication between home and school* Parents believed that they had insights to offer school, in line with the logic model component of ‘collaboratively share knowledge and expertise’: *“I think it would have been helpful in terms of them knowing what we do at home, what helps or doesn’t help, and also how her ADHD presents, and what she struggles with. Because I think they just see the small bits, in terms of her struggling to focus, but I’m not sure they see the bits that lead up to that.”*

Some parents described being *“a little bit in the dark”* and in some cases were critical *“there is no communication [from school] whatsoever”*. However, most parents felt that schools’ engagement with the research study and the toolkit was an implicit demonstration of their awareness of their child’s additional needs, and appreciated this. Parents had many ideas and varied preferences as to how this could best be done, with some preferring being spoken to in person before or after school, at parents evenings, and others appreciating contact by email or by phone.

*Teacher collaboration.* Teachers reported being used to working collaboratively with groups of peers (and unused to working alone on a new intervention). Teachers in the study consistently reported that the toolkit could be better integrated into their routine if they were working with a small group of others (or the whole school) as they could set targets together, share ideas, and help to hold each other accountable. Perspectives on this varied, with some suggesting that all teachers use the same strategy each week, whereas others suggested using the toolkit individually but being able to share ideas and get feedback from one another.

*Study team involvement.* Overall, teachers did not feel the need to reach out to the study team. However, they did report clear awareness of the lines of communication (phone, email) and were clear that they felt they could contact the study team if they needed to. One or two had difficulties with their website login, and reported positively on their interactions with the team to resolve this. Other teachers raised points of contention with their school during their process evaluation interview, and were highly receptive of the addition of an extra visit to support them to select and prepare to implement resources. This was a difficult judgement for teachers to make as they explained that their priority was to be with their class, teaching, during the school day as much as possible. As such, they did not want more time out of their classroom unless another qualified teacher was providing cover, but understood the need for this and felt that 15 minutes was achievable and not overly detrimental to their teaching delivery. Both parents and teachers considered all communication from the study team to be useful and appropriate, but communication between home and school regarding the toolkit was much more varied (see contextual factors).

**Questionnaire feedback**

Teachers believed that a maximum of one set of questions each half term would be most appropriate. They discussed how they experienced worse behaviour from all children at the start and end of a school term, indicating that future questionnaires should be consistent in terms of timing across the school term. They appreciated reminders sent by the study team, which they considered to be friendly and appropriate. Teachers reported that if they did not complete a questionnaire as soon as they saw the initial email, they were unlikely to remember without further reminders due to their cognitive load. Some reported enjoying completing the questionnaires as this gave them space to reflect on an individual child, considering them “*worthwhile*”, and several reported that the repeated measures got easier with time. The long questionnaires (taking 15-20 minutes versus 4-5 minutes for short questionnaires) were considered the most burdensome part of the study overall; teachers reported feeling “*guilt*” when they had not completed these, and were sometimes unable to do these without additional cover provided for their class. Cover arrangements varied widely by school, with some SENCos providing cover for all staff to complete every long questionnaire, but others unable to do this for logistical reasons. Other challenges experienced by teachers and parents in completing questionnaires were the variability of children’s behaviour over time. As such, the timeframe that is asked about needs to be carefully considered, as children’s behaviour could vary daily, weekly or even moment-to-moment.
